# Practical Guidance on Selecting Analytical Methods for PFAS in Semiconductor Manufacturing Wastewater

**DOI:** 10.1021/acsmeasuresciau.5c00021

**Published:** 2025-06-20

**Authors:** Boris Droz, Christopher G. Heron, Mitchell L. Kim-Fu, Patrick N. Reardon, Mireia Roig-Paul, Jennifer A. Field

**Affiliations:** † Department of Biological and Ecological Engineering, 2694 Oregon State University, Corvallis, Oregon 97331, United States; ‡ Department of Environmental and Molecular Toxicology, Oregon State University, Corvallis, Oregon 97331, United States; § Department of Chemistry, Oregon State University, Corvallis, Oregon 97331, United States; ∥ NMR Facility, Oregon State University, Corvallis, Oregon 97331, United States

**Keywords:** semiconductor, wastewater, per and polyfluoroalkyl substances (PFAS), high-resolution mass spectrometry (HRMS), gas chromatography mass spectrometry (GC–MS), ultrashort-chain PFAS, total organic fluorine, nuclear magnetic resonance (NMR)

## Abstract

The focus of this review is to provide an overview of the nomenclature, structure, and properties of perfluoroalkyl and polyfluoroalkyl substances (PFAS) that dictate the selection of analytical methods for analyzing PFAS in treated semiconductor wastewater. The review is organized by introducing the fundamental concepts of how structure dictates the physical-chemical properties of PFAS and how these properties determine the suitability and applicability of standardized analytical methods for individual PFAS as well as methods for total fluorine. Structures for PFAS measured in semiconductor wastewater or known to be in use by industry are given with tables intended as guidance for method selection. This review includes current guidance on sample collection, storage, and handling along with a comparison of U.S. Environmental Protection Agency and American Society for Testing and Materials analytical methods for target PFAS as well as methods for ultrashort PFAS. Methods are reviewed for volatile PFAS in wastewater as well as workflows for suspect and nontarget nonvolatile and volatile PFAS. Nonspecific methods for PFAS including the total oxidizable precursor assay, total fluorine analyses, and extractable and adsorbable organic fluorine assays are reviewed. Alternative detectors for total fluorine are reviewed along with nuclear magnetic resonance spectroscopy and sensors for online wastewater monitoring.

## Graphical abstract

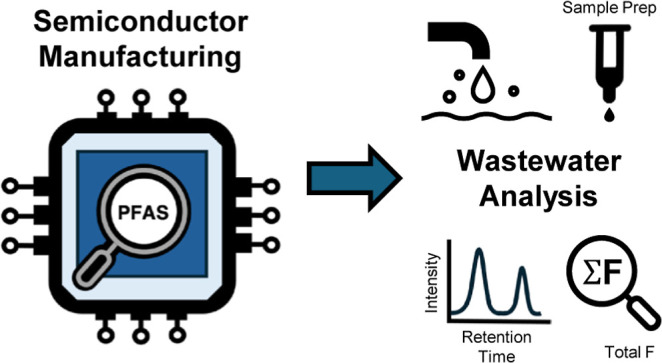

## 1 Introduction

The semiconductor industry is integral to the global economy. The industry employs a variety of physical and chemical processes, using chemicals that are necessary to generate high-performance products that are vital for many electronics. The nature of per- and polyfluoroalkyl substances (PFAS), including a number of carbon–fluorine bonds with strong electron withdrawing regions, makes them resistant to chemical and thermal degradation. Many PFAS provide a range of distinctive properties (e.g., superacid, low surface energy, low refractive index, and low dielectric constant) that allow for accurate and reliable production of semiconductors.^1–3^

The Semiconductor PFAS Consortium has released a number of technical papers outlining the myriads of roles that PFAS play in the semiconductor industry.^2–12^ The Consortium utilizes a working definition of PFAS as any chemical with a C–F_2_ or C–F_3_ group.^2^ This definition is consistent with the Organisation for Economic Co-operation and Development (OECD) definition,^13^ which is more inclusive than earlier definitions,^14^ and ensures the Consortium’s efforts encompass all potentially regulated forms of fluorine-containing chemicals. However, this definition does not signify the Consortium endorsement of one definition over another. Greenhouse gases and fluoropolymers are excluded from this review, although these are also used by the semiconductor industry.^4,5,8,15^

A growing body of literature describes the semiconductor industry as a source of PFAS emissions.^16–21^ To date, there are no convenient guides that compile PFAS known to be used by the semiconductor industry, either through patent searches or expert knowledge or from measurements of PFAS in treated semiconductor wastewater. Discovering molecular structures is a time- and resource-intensive endeavor; thus, it is much more expedient to know the structure of PFAS used by the semiconductor industry, since structure guides the selection and optimization of analytical methods. Likewise, analytical methods can provide insight into PFAS behavior such as the potential for oxidation and sorption. Several excellent reviews exist on subtopics covered by this review, including analytical methods that cover a wide range of individual PFAS^22^ and nonspecific PFAS methods; however, the focus of previous reviews is on meeting the needs of the semiconductor industry.^23,24^ The overarching goals of this review are to highlight methods for analysis, with their advantages and limitations, for PFAS in treated semiconductor wastewater and provide reference guides for selecting analytical methods for detecting the potentially wide range of PFAS in treated semiconductor wastewater.

The review consists of ten sections that serve as a primer and overview on PFAS, the methods available for analysis, and their relevance to semiconductor wastewater:

1 PFAS structure governs properties
2 PFAS used in the semiconductor industry with potential to occur in wastewater
3 Water sample collection, storage, and handling
4 Methods for target nonvolatile PFAS
5 Methods for ultrashort-chain PFAS
6 Volatile target PFAS methods
7 Suspect and nontarget PFAS workflows for high-resolution mass spectrometry
8 Nonspecific methods for PFAS analysis
9 ^19^F NMR for quantitative and qualitative PFAS analysis
10 Sensors for online monitoring of PFAS in wastewater.

This review offers an overview of the key structural aspects of PFAS and a guide to selecting analytical methods based on the PFAS structure. Target PFAS (Tables S1 and S9) are PFAS for which analytical standards are currently commercially available, while suspect PFAS (Tables S2–S6) are those that have been previously identified but for which there are no commercial analytical standards and nontarget PFAS which refer to unidentified PFAS. For the purposes of this review, standardized methods available in the U.S. for the analysis of target PFAS in water including U.S. Environmental Protection Agency (EPA) Method 1633,^25^ 537,^26^ 537.1,^27^ 533,^28^ 3512,^29^ and 8327 ^30^ are reviewed along with ASTM International (ASTM) Method D7979-20 ^31^ for their applicability to PFAS associated with treated semiconductor wastewater. In addition, methods for ultrashort chain PFAS are reviewed. Reference guides in the form of tables present in Supporting Information allow users to quickly identify which methods are applicable in their current form or could potentially be modified to include suspect (previously identified but with no analytical standard) or nontarget (previously unidentified) PFAS in wastewater. Workflows for the detection of volatile suspects and discovery of nontarget PFAS are also discussed together with nonspecific analytical methods and alternative detectors for total fluorine. While no universal methods exist for volatile PFAS in wastewater, published methods for target, suspect, and nontarget volatile PFAS as well as instrumental considerations are discussed. The utility of nuclear magnetic resonance spectroscopy (NMR) is discussed for its applicability for PFAS detection in wastewater. A review of the status of sensor for online methods for PFAS detection in wastewater will be addressed. For all methods, their advantages and limitations are presented as part of the guidance with a convenient “stoplight” approach for selecting analytical methods for PFAS detection in semiconductor wastewater. The semiconductor industry uses PFAS that range from highly volatile fluorinated greenhouse gases, including hydrofluorocarbons and perfluorocarbons, to higher-molecular weight volatile PFAS (e.g., fluorotelomer alcohols (FTOHs)), to ionogenic PFAS that are capable of ionizing in water as a function of pH, to higher-molecular weight polymer-bound PFAS, and fluoropolymers (Figure 1). The range of chemical properties associated with PFAS is wide and includes vapor pressure, aqueous solubility, ionizability, and tendency to partition to solids (e.g., sorbents). Knowledge of the chemical properties must be considered when selecting approaches for sample concentration, analyte separation, and detection. For example, fluoroalkanes and other volatile PFAS typically require gas chromatography (GC)-based approaches, while ionogenic PFAS require liquid chromatography (LC)-based approaches. Together, GC and LC with mass spectrometry (MS) are the most commonly used analytical separation and detection method. The choice of the method used to cover a range of PFAS generally requires more than one analytical method, with some overlap between methods. For the purpose of this review and brevity, semivolatile PFAS will be hereafter termed volatile PFAS unless otherwise noted. High-molecular weight, polymer-bound PFAS and fluoropolymers require alternative methods (Figure 1). For the purposes of this review, the focus is on volatile and nonvolatile PFAS.

**Figure 1:**
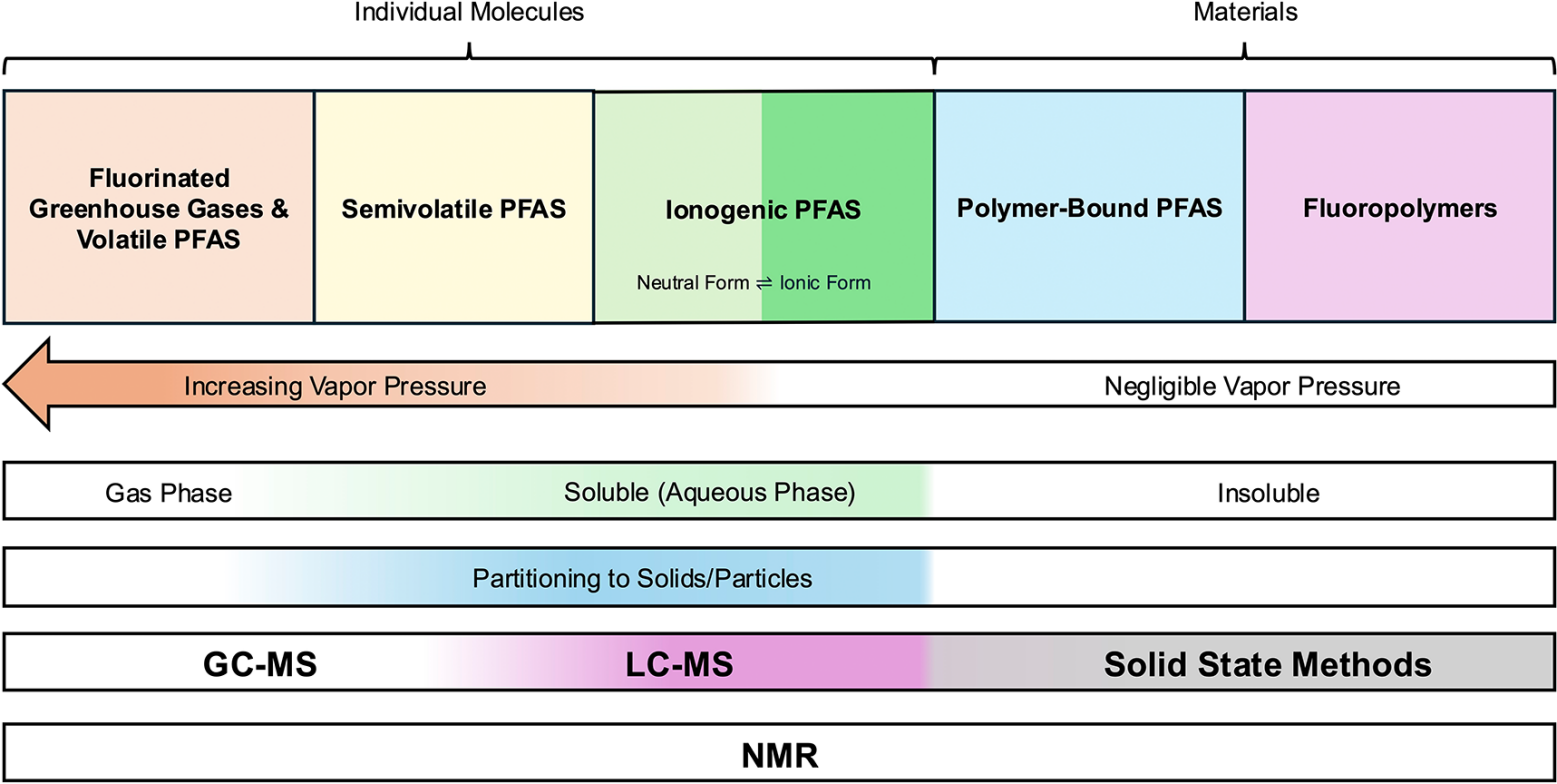
Range in chemical properties of PFAS associated with semiconductor activities.

## 2 PFAS Structure Governs Properties in Semiconductor Wastewater

The nomenclature of PFAS has evolved over time to categorize PFAS.^14,32,33^ Perfluorinated substances are those in which all of the hydrogens on carbons are replaced by fluorine. Relatively few PFAS subclasses meet this definition, and these subclasses include perfluoroalkyl sulfonates (PFSAs), perfluoroalkyl carboxylates (PFCAs), perfluoroalkyl sulfonamides (FASAs), and perfluoroalkyl ether acids (PFEAs), as well as perfluoroalkyl fluorides, iodides, or aldehydes.^32^ Each subclass has a unique headgroup but may have one or more homologues that vary by their fluorinated or fluorinated polyether chain length. Polyfluorinated PFAS make up the remaining myriad of subclasses in which the fluorinated chain contains carbons that are not fully fluorinated. However, as the diversity of PFAS structures increases, alternative approaches are being developed to classify PFAS using their structural elements.^33^ In this review, it is necessary and convenient to specify a specific carbon chain length when referring to a PFAS. In this case, the number of carbons specified refers to the total number of carbons and not the number of fluorinated carbons. For example, the C4 PFCA, perfluorobutanoic acid (PFBA), has three fluorinated carbons and one nonfluorinated carbon. In contrast, PFAS synthesized via fluorotelomerization, such as the 6:2 FTOH, would be referred to as the C8 FTOH because it has six fluorinated carbons and two nonfluorinated carbons. The “*n*/*m*” nomenclature, associated with fluorotelomerization, represents the number of fluorinated carbons (*n*) and the number of nonfluorinated carbons (*m*).

The length of the fluorinated chain ranges from ultrashort- (e.g., C1–C3)^34–38^ to longer-chain PFAS (e.g., C8–C14) and higher.^32^ In the case of polyether compounds, the fluorinated chain is broken into smaller segments by oxygens, such as HFPO–DA (e.g., GenX) and 4,8-dioxa-3*H*-perfluorononanoic acid (ADONA).^39^ The diversity among PFAS subclasses is determined by the head groups. The most well-known head groups are those with carboxylate (e.g., PFCAs), sulfonate (e.g., PFSAs), and sulfonamide (e.g., FASAs) head groups.

The key to selecting analytical methods for PFAS analysis is understanding their structure and how that structure governs their properties. Chemical structure governs speciation (e.g., charged state) of any molecule. Many PFAS are ionogenic (the ability to become ionized), where the charged state determines key physical-chemical properties, including solubility, volatility, and hydrophobicity, that, in turn, inform the sample preparation and detection methods for that molecule.^40^

Many PFAS used in the semiconductor industry are sold in salt forms, where anionic PFAS are paired with counter cations such as potassium, ammonium,^12^ or as more complex organic sulfur- or iodine-containing onium cations.^1,3^ However, once in contact with water, the salts of ionogenic PFAS dissociate into their respective ions,^32^ and the PFAS will speciate according to their acid-dissociation constant (p*K*_a_).^40^ Likewise, any PFCAs and PFSAs sold in their free acid (protonated forms) will also dissociate in water to form H^+^ and the PFCA or PFSA anion. As very strong acids with very low p*K*_a_ values, PFCAs and PFSAs are present in their anionic forms at all environmental pH values. In contrast, perfluorobutane sulfonamido ethanol, which is sold to the semiconductor industry as an alternative to perfluorooctanesulfonic acid (PFOS),^12^ has a very high p*K*_a_ (∼12–14) and occurs in wastewater in its un-ionized, neutral form. What is interesting and perhaps unique to the semiconductor industry is the strongly acidic to basic conditions required for many fabrication processes. There may be stages within semiconductor fabrication systems where the pH of the waste stream is low or high relative to the p*K*_a_ of PFAS that will impact the speciation of ionogenic PFAS and therefore the PFAS physical-chemical properties (Figure 1). For example, a significant fraction of FASAs may be in the neutral, volatile form at low pH but in their anionic, nonvolatile form at a higher pH.

Many well-known PFAS are ionogenic where the speciation in wastewater to charged or neutral forms is a function of the headgroup’s p*K*_a_ and the pH of water (e.g., wastewater). Unfortunately, few p*K*_a_ values of PFAS have been measured. Proximity of the ionogenic headgroup to the fluorinated carbon chain determines the magnitude of their p*K*_a_. Although there are few experimental values for PFCAs,^41,42^ recent modeling efforts indicate the p*K*_a_ values of PFCAs in water are less than one and that the fluorinated chain length has a limited effect on the magnitude p*K*_a_ values.^43^ While the PFSA subclass is considered a super acid,^1^ many strong acid PFAS subclasses are anionic at the pH of wastewaters (e.g., 5–8) given their low p*K*_a_ values.^41,44,45^

There are a number of weak acid PFAS classes, including the unsubstituted (FASA), substituted *N*-methyl (Me), and *N*-ethyl (Et) FASAs (e.g., *N*-Me-perfluorooctane sulfonamide (FOSA), *N*-Et-FOSA) with estimated p*K*_a_ values near neutrality.^46^ The sulfonamido acetic acid forms, including Me-perfluorooctane sulfonamido acetic acid (FOSAA) and Et-FOSAA (Table S1), are weak acids since the carboxylic acid groups are not bonded directly to fluorinated carbons. Cationic PFAS are those with a fixed positive charge (e.g., quaternary amines) or have ionogenic nitrogen atoms that become cationic at pH values according to their p*K*_a_.^47^ In addition, there are many subclasses of zwitterionic PFAS that possess two or more ionogenic head groups.^47–51^ However, to date, there are no reports of cationic or zwitterionic PFAS used by the semiconductor industry.

The properties of ionogenic chemicals are a function of their speciation with higher aqueous solubilities for charged (e.g., negatively or positively) species compared to their neutral forms.^40^ Data on the aqueous solubility of PFAS are available but are for specific salts of PFAS such as PFOS and perfluorooctanoic acid (PFOA).^52,53^ Charged species exhibit very low vapor pressure and are generally considered nonvolatile; however, if present in air, they are likely associated with particulate matter or aerosol fraction.^54^ Some neutral volatile PFAS have higher vapor pressures and are associated with the gas-phase fraction in air.^54^ Care must be taken when selecting properties without accounting for speciation of the molecule of interest. For example, the vapor pressures of the free acid form of ionogenic PFAS such as PFCAs and PFSAs^55^ will not apply to semiconductor wastewater at neutral pH since PFCAs and PFSAs will be in their anionic form and not in their protonated, free acid forms. Although not covered in this review, additional partitioning properties of PFAS including the air–water partition coefficient (Henry’s law) and organic carbon–water partition coefficients are impacted by speciation, with neutral species partitioning into air and exhibiting greater partitioning to the organic carbon fraction of soils and sediments.^40^ Cationic and zwitterionic PFAS exhibit greater association with negatively charged environmental solids.^50,56–63^

Volatile PFAS are those with head groups that do not ionize in the environmental pH range of wastewater (Table S2). Subclasses including FTOHs and substituted perfluoroalkyl sulfonamido alcohols (FOSE), Me- and Et-FOSE, have very high p*K*_a_ values, such that they occur in their neutral forms at environmental pH values (note the proton is not red in Table S1 since it is not ionogenic at environmental pH values (4–10)).^40^ Other head groups on target volatile PFAS include acrylates, methacrylates, olefins, iodides, acetates, ketones, and aldehydes. In addition, ionogenic perfluorooctane sulfonamide (FOSA), Me-FOSA, and Et-FOSA with p*K*_a_ values near the pH of wastewater will speciate such that their neutral and ionized forms are present (Table S2).

## 3 PFAS Used by the Semiconductor Industry with Potential to Occur in Semiconductor Wastewater

At present, there are few published papers that document the measurement of nonvolatile PFAS in treated semiconductor wastewater.^16,17,19^ There are some peer-reviewed papers that describe PFAS in surface waters that are downstream from semiconductor facilities but may be attributable to more than one source.^18,20,21,64^ To the best of our knowledge, there are no papers that measure volatile PFAS in treated semiconductor wastewater. In addition, publications that list PFAS associated with the semiconductor industry from an expert point of view and patent analysis but lack data on occurrence in treated semiconductor wastewater are presented in Tables S4–S6.

Treated semiconductor wastewater is defined for this review as wastewater treated onsite using physical, chemical, and/or biological treatment to meet national, local, and company-specific effluent discharge requirements, before discharge to local wastewater treatment facilities or surface water.^65^ The typical treated semiconductor wastewater effluent ranges in characteristics such as pH 6–11,^65^ fluoride ion concentrations (0.66–100 mg/L), and PFAS concentrations in the ng/L to μg/L range.^65^ However, additional characteristics such as ionic strength, organic solvents, and oxidant concentrations in treated semiconductor wastewater that may affect analytical method performance are not well documented and may vary between semiconductor facilities.^65^ Although effluents may vary in their aqueous chemistry, the matrix is likely analogous to other municipal and industrial wastewater effluents. The methods for sampling and analysis of PFAS in this review were developed for matrices other than treated semiconductor wastewater, such as surface water and groundwater. However, wastewaters discharged from fabrication unit processes (tools) may vary more widely in pH, ionic strength, organic solvent, oxidant, and PFAS concentrations compared to more dilute, treated semiconductor wastewater effluent. Therefore, for many of the sampling and analysis protocols, the performance of the analytical methods for treated semiconductor wastewater effluent and wastewater effluent coming from fabrication unit processes is unknown and represents a research need.

The PFAS reported in semiconductor wastewater in the U.S. by Jacob et al.^19^ are a combination of target PFAS (Table S1) including PFCAs (C4–C10) and perfluorobutanesulfonic acid (PFBS) and mostly strong-acid suspects including carboxylates, acidic alcohols, ether-, and polyether-based PFAS (Tables S2 and S3) that were discovered through nontarget workflows. The shortest chain length evaluated included PFBA and PFBS (Table S1); ultrashort-chain PFAS (<C4) were not evaluated in this study. Extraction from wastewater was accomplished using solid phase extraction (SPE) with the capacity for acidic, basic, and neutral PFAS, followed by reverse-phase separation and detection in negative mode by HRMS.^19^ Due to detection being in negative mode, no PFAS with positively charged head groups were detected, while estimated concentrations of suspects exceeded that of target PFAS.^19^ Many of the suspects are composed of carboxylate and very acidic alcohol head groups (OH group bonded to fluorine-bearing carbon), while others are ether- and polyether-based structures (Table S2). Jacob and Helbling^66^ performed a second study in which they reported ultrashort-chain PFAS in semiconductor wastewaters, including trifluoroacetic acid (TFA), pentafluoropropionic acid (PFPrA), trifluoromethanesulfonic acid (TFMS; also known as triflate in the semiconductor industry),^1^ and perfluoropropanesulfonic acid (PFPrS).

Chen et al. reported target and suspect PFAS in treated semiconductor wastewater^16,17^ and in downstream river water in Taiwan.^18^ Analyses were conducted using weak anion exchange (WAX) SPE with detection in negative mode by HRMS and a workflow that focused on molecules that gave fragments unique to PFAS. No analyses were conducted to determine if PFAS with positively charged head groups were present. Target PFAS included PFCAs (C4 and C14) and PFBS (Table S1). Suspect PFAS found were largely composed of four fully fluorinated carbons and were detected in negative mode due to their anionic carboxylate, sulfinate (e.g., –SO_2_^–^), and substituted sulfonamide headgroups (Table S3). However, they also reported the presence of ultrashort-chain PFCAs including TFA, PFPrA, and TFMS. Disubstituted sulfonamido ethanols were also detected (class 8; Table S3) even though they are unlikely to be anionic in wastewater. Thus, it is likely that they were extracted in their neutral form and detected as adducts in negative mode. It is interesting to note that while both groups used nontarget workflows, they found very different classes of PFAS in their wastewater samples, which may reflect the PFAS chemical market in the U.S. versus Taiwan. Finally, a lack of data exist on a number of categories of PFAS that make up significant fractions of the PFAS used by the semiconductor industry, including water-soluble fluoropolymers (17,182 kg/yr or 50.9%).^67^

## 4 Water Sample Collection, Storage, and Handling

Given the unique properties of PFAS, several studies have provided overviews on issues surrounding sampling collection techniques, sample storage conditions, and steps taken prior to analysis that may be the source of negative or positive artifacts.^22,68,69^ This section is organized into sampling wastewater, sampling materials, sample container types, storage considerations, and filtration. The storage and stability characteristics for the suspect PFAS reported by Chen et al.^16,17^ and Jacob et al. have not been determined, in part due to the lack of standards available to conduct experiments.^19^ To increase confidence in novel PFAS identified in semiconductor wastewater, high quality analytical standards and stable isotope-labeled standards will be needed.

### 4.1 Sampling Wastewater

For certain chemicals in wastewater that exhibit short-term temporal variability, grab samples are not the most appropriate approach for obtaining representative samples, especially if the end goal is to estimate mass flows (mass/day).^70,71^ However, if the goal is to establish the temporal variability in a given chemical’s concentration, grab samples are appropriate. Given that there is limited data for PFAS in semiconductor wastewater, and even less is known about the temporal variability in the PFAS composition of semiconductor wastewater, sampling campaigns should use recommended techniques for obtaining representative samples of wastewater, depending on the question being asked.^70–74^ One of the alternatives for sampling wastewater is passive sampling, which obtains a time-weighted average of the freely dissolved concentration of a chemical over an extended period of time.^75,76^ Consequently, this approach has received increasing attention for its application to PFAS.^75–81^ At present, the variability in the PFAS composition of semiconductor wastewater over time is unknown and may vary by the facility. However, average semiconductor wastewater effluent flow (m^3^/day) is reported^65^ and can be used to compute PFAS mass flows as PFAS concentration data become available. Thus, composite sampling or passive sampling may more accurately capture the PFAS mass flows in semiconductor wastewater.

### 4.2 Sampling Materials

Two studies report PFAS associated with sampling materials used in the field. Rodowa et al.^82^ examined sixty-six materials for 52 PFAS, but only 22 materials had the potential to come in direct contact with water samples, and none of the 22 gave quantifiable PFAS concentrations. Leaching of PFAS from polytetrafluoroethylene and low-density polyethylene (LDPE) tubing, bailer lines, and water level tape into deionized water was reported after 24 h of soaking the articles in deionized water.^83^ However, a 24 h contact time with these materials is unlikely for field samples under typical sampling conditions (temperatures above 4 °C).^83^ Despite these reports of PFAS associated with materials used in the field, there are few plausible pathways for the PFAS to cross-contaminate a water sample unless the materials come into direct contact with the water sample. Given these findings, it may be unnecessary to place strict limitations on materials used in the field, such as reusable ice pack, but it remains prudent to eliminate materials composed of fluoropolymers that come into direct contact with water samples.^68,69,82^ With increasing instrument sensitivity and lower acceptable levels of PFAS in water, this topic should be revisited.

There is a growing body of literature that provides data to support the most appropriate choice of bottle type for sampling PFAS in (waste)­water.^84^ Woudneh et al.^85^ and others^86,87^ demonstrated most loss of PFAS, particularly long-chain, from water when stored in glass and polypropylene compared to high-density polyethylene; however, when the bottle was rinsed with methanol, the PFAS were recovered. Whole bottle analysis is part of U.S. Environmental Protection Agency (EPA) Method 1633.^28^ Zenobio et al. also reported loss of all PFAS from water onto six types of container materials with greater losses for long-chain PFAS and for PFSAs compared to PFCAs and for sulfonamides.^84^ Lenka et al. reported up to 20% losses of short-chain PFBS, PFBA, and PFPrA to polypropylene samples tubes.^88^ The most appropriate type of sample container and the need for whole-bottle analyses should be confirmed for the potentially large spectrum of PFAS found in treated semiconductor wastewater.

### 4.3 Storage Conditions

Sample storage temperatures and hold times were examined by Woudneh et al.^85^ They reported the formation of select PFCAs, FOSA and Me- and Et-fluoroalkyl sulfonamido acetic acid (FOSAA) and degradation of Me- and Et-FOSE to Me-FOSAA and Et-FOSAA, respectively, over 14 days of storage at 4 °C in waters including wastewater and surface water. Thus, Woudneh et al.^85^ recommend storage of wastewater and other environmental waters at −20 °C. The stability of PFAS during storage in solvents was investigated by Zhang et al.^89^ They found that while PFCAs, PFSAs, and *n*:2 FTS were stable when stored at room temperature in deionized water, methanol, or isopropyl alcohol over 30 days, polyfluoroalkyl ether acids degraded with increasing temperature and with decreasing water-to-organic solvent ratio. The findings of Zhang et al. indicate that, over time and with regard to storage, care must be taken when making analytical measurements of polyfluoroalkyl ether acids,^89^ which are reported for semiconductor wastewater.^19^

### 4.4 Filtration

There is a significant body of work that demonstrates the loss of PFAS during the filtration of water, and there is a growing consensus about avoiding filtration to prevent false negatives through loss from solution.^90–93^ Loss of PFAS occurs for a wide variety of reasons, including losses to filters for PFAS as chain-length increases.^90–92^ Some reports indicate filtration losses are caused by headgroup effects,^92^ while others report little effect of the headgroup.^90–92^ Although filtration did not impact the recovery of target PFAS from semiconductor wastewater,^19^ additional research is needed to understand potential effects of filtration on suspect and nontarget PFAS.^23^

## 5 Methods for Target Nonvolatile PFAS

### 5.1 U.S. EPA and ASTM Methods

The U.S. EPA currently has four PFAS methods available for target PFAS in aqueous matrices (Table S1). These methods focus on anionic PFAS, with the exception of Method 1633,^25^ which includes two neutral FOSEs. At present, Method 6133 does not include neutral FTOHs. U.S. EPA Methods 533,^28^ 537,^26^ and 537.1 ^27^ were designed and validated for drinking water, which is a relatively clean matrix with few interferences, while U.S. EPA Methods 1633,^25^ 3512 ^29^ (extraction) used in combination with 8327 ^30^ (analysis), and ASTM D7979–20,^31^ ASTM D8421,^94^ ISO 21675:2019,^95^ and DIN 38407 ^96^ are for matrices other than drinking water, such as wastewater (Table S7). Methods 537 and 537.1 were available prior to the wastewater specific methods.

Method 537 was the original U.S. EPA method for PFAS in drinking water and included 14 target PFAS among PFCA, PFSA, FASA, and substituted FASA classes.^26^ Method 537.1 is intended for finished drinking water, which is a simple sample matrix with potentially few interferences (Table S7).^27^ When Method 537 was executed, the method stipulated that it must be performed as written without modification. Method 537 was updated to Method 537.1 to include a total of 18 PFAS (Table S1).

### 5.2 Isotopic Dilution

Both Methods 533 and 1633 utilize isotopic dilution. Isotopic dilution is a quantitation scheme where stable isotope-labeled PFAS standards are added prior to sample extraction to compensate for native (nonlabeled) PFAS loss that occurs during extraction and analysis (Table S7). A small subset of PFAS has a second isotopically labeled version, which is typically added after sample preparation steps and is used to quantify the recovery of the first stable isotope added prior to extraction. Within the various EPA and ASTM methods, the nomenclature associated with the stable isotope PFAS standards vary. For the purposes of this review, the first stable isotope added prior to extraction is termed MPFAS, while the second stable isotope is termed M2PFAS. An example of a MPFAS is ^13^C_4_-PFOS, and the M2PFAS is ^13^C_8_-PFOS.

The behaviors of the native PFAS and their MPFAS and M2PFAS analogues regarding extraction and chromatographic separation are equivalent. However, their masses are sufficiently different such that mass spectrometers can distinguish the native PFAS, MPFAS, and M2PFAS. The ratio between a native PFAS and its MPFAS is then used to calculate the native PFAS concentration and accounts for any loss of the native PFAS. For target PFAS that do not have matching MPFAS, a MPFAS is selected based on how closely it matches the target PFAS in terms of functional headgroup and chromatographic retention time. The recovery of MPFAS is determined as the ratio of MPFAS to M2PFAS. Stable-isotope dilution is a powerful tool when used in analytical methods for PFAS in complex matrices such as wastewater. The price of MPFAs and M2PFAS adds to the analytical costs. However, despite its limitations, isotope dilution is considered to be the best practice for determining PFAS concentrations in wastewater and other environmental media.

Method 537.1, which does not employ isotope dilution, provides PFAS concentrations that do not consider any losses during sample preparation and thus may underestimate PFAS concentrations. Method 533 is an isotopic dilution method for 25 target PFAS, 16 MPFAS, and three M2PFAS (Table S7).^28^ Like Method 537.1, Method 533 is intended for finished drinking water; however, due to the fact that it utilizes isotope dilution, Method 533 is more suitable for matrices other than drinking water. Since Method 537 was replaced by Method 537.1, Method 537 is not discussed further.

Method 1633 utilizes isotope dilution for the analysis of 40 target PFAS in a multitude of matrices, including groundwater, surface water, wastewater, soil, sediment, landfill leachate, and fish tissue.^25^ Of the 40 target PFAS, 24 have matched MPFAS, and seven have matched M2PFAS. The more matched MPFAS that are used translates to higher confidence in the accuracy of the reported target PFAS concentrations in complex aqueous matrices such as wastewater. In January 2024, Method 1633 was finalized though a multilaboratory validation study that developed statistically derived quality control limits.^97^ With the final version of Method 1633, laboratories may now modify it and add new PFAS as standards become commercially available, as long as the method performance is within quality control limits.^25^ International methods that similarly utilize isotopic dilution and solid phase extraction (SPE) are ISO 21675:2019 ^95^ and DIN 38407-42.^96^ U.S. EPA Method 3512 ^29^ (extraction) is used in combination with US EPA Method 8327 ^30^ (analysis) as well as ASTM D7979-20,^31^ and is designed for nondrinking water matrices, such as wastewater. Methods 3512 and 8327 are used together for 24 target PFAS and 19 MPFAS, where Method 3512 pertains to the samples preparation,^29^ and Method 8327 pertains to the analysis.^30^ These methods do not require SPE and are thus faster and less expensive to perform. Method 3512 and the international method ASTM D842 utilize solvent dilution of a wastewater matrix prior to injection onto an instrument instead of SPE.^29^ The ASTM D7979-20 is an international method that utilizes direct injection (no sample preparation) for 21 target PFAS and nine MPFAS.^31^ Both the EPA 3512/8327 and ASTM D7979-20 methods utilize MPFAS to assess the method performance and accuracy (Table S7). The advantages of U.S. EPA Method 3512/8327 and ASTM D7979-20 are quick turnaround times and low cost. However, disadvantages include the use of external calibration, which does not account for instrumental variability and relatively high reporting limits compared to SPE methods, due to the lack of sample concentration. Recent data on PFAS concentration on semiconductor wastewater were acquired using a modified version of EPA Method 537.1 for wastewater, EPA Method 1633, and ASTM 7979-20.^65^

### 5.3 Sample Preparation

U.S. EPA Methods 537.1,^27^ 533,^28^ and 1633 ^25^ and international methods ISO 21675:2019 ^95^ and DIN 38407-42 ^96^ use solid phase extraction (SPE). SPE is a process where water is passed through a sorbent-filled cartridge. Aqueous matrices much as wastewater are passed through SPE sorbent cartridges, thus allowing sample concentration. Sorbed PFAS are eluted, and the extract is further concentrated to achieve lower detection limits. There are many different types of sorbents available, and the type of sorbent selected determines the mechanism of interaction for concentrating PFAS and thus the classes of PFAS concentrated. At present, three types of SPE sorbents are primarily used for PFAS extraction. Method 537.1 utilizes a reverse-phase sorbent where PFAS are concentrated onto the sorbent according to their hydrophobicity.^27^ A more widely used SPE sorbent is WAX, which is used in Method 1633.^25^ By controlling the pH of the sample, the WAX sorbent adopts a positive charge, thus concentrating anionic PFAS by electrostatic interactions (e.g., ion exchange). Compared with reverse-phase sorbents, WAX sorbents are more selective at removing anionic PFAS from a matrix and generally give better analytical performance (accuracy and precision). In the case of WAX SPE, its suitability for other neutral, cationic, or zwitterionic PFAS has not been thoroughly evaluated, although neutral FOSEs are determined by Method 1633 and ASTM D8421. Method 533 uses mixed-mode SPE, which is a hybrid that includes both reverse phase and WAX properties.^28^ For a wide range of anionic PFAS, WAX SPE retains the widest range of PFAS based on functional group.^98^

### 5.4 Mass Spectrometric Detection

For the analysis of target PFAS (Table S1), separations are performed by utilizing liquid chromatography followed by detection by mass spectrometry. The most common interface for anionic PFAS is an electrospray ionization interface (ESI), since most target PFAS are “preformed” ions or are easily ionized. An alternative ionization interface, atmospheric pressure chemical ionization (APCI), is not commonly used for PFAS analysis as it ionizes poorly ionized compounds but yields higher background. Most analytical methods for target PFAS use a triple quadrupole or “tandem” mass spectrometer (i.e., LC–MS/MS). The LC–MS/MS has the advantage of being highly sensitive, allowing for low levels of detection and robustness at a relatively low instrument price. The LC–MS/MS uses a mass filter to select a precursor (unfragmented) ion, which is then passed into a collision chamber where the precursor ion is fragmented into product ions. Multiple reaction monitoring (MRM) consists of filtering the precursor ions, fragmenting them, and filtering the resulting fragments, resulting in a high degree of confidence in the identity of the PFAS detected. Confidence in the identity of the PFAS detected is increased when multiple fragments are produced by a precursor ion, where the most abundant (based on area count) fragment ion is used for quantification of the target PFAS, while the next most abundant ion is used as the qualifier ion used to confirm the target PFAS identification. The area counts of the quantification and qualifier ions are ratioed and are required to match that of PFAS standards; deviations from the expected ratio indicate the presence of an interference that can cause a false positive.

### 5.5 Implications for Semiconductor Wastewater

The best method for wastewater analysis depends on the analyte concentrations and class of analytes tested. For trace level analysis of anionic PFAS, an isotopic dilution method utilizing whole bottle extraction yields the most accurate measure of the PFAS concentrations. The “industry standard” is rapidly becoming the US EPA Method 1633. This method is designed for wastewater and is expandable to any anionic and potentially some neutral PFAS for which analytical standards exist. The downside of this method is its cost and the focus on PFAS that interacts with WAX SPE during extraction. For the screening of all neutral, anionic, cationic, or zwitterionic compounds at higher microgram per liter or higher concentrations, either U.S. EPA Methods 3512 ^29^/8327 ^30^ or ASTM D7979-20 ^31^ or ASTM D8421 ^94^ is recommended. These methods potentially capture all forms of PFAS at a significantly lower cost; however, they should only be applied if concentrations reaching microgram per liter or higher of PFAS of interest are expected.

## 6 Methods for Ultrashort-Chain PFAS

The occurrence of ultrashort-chain PFAS in semiconductor wastewater was recently demonstrated.^17,66^ Ultrashort chain PFAS are defined as those PFAS with less than four fluorinated atoms.^66,99^ The hydrophilic nature of ultrashort-chain PFAS presents analytical challenges, namely, their extraction from water and subsequent ability to focus and separate them chromatographically. Therefore, analytical method development for ultrashort-chain PFAS focuses on optimizing SPE media for their concentration from water along with selecting analytical columns for focusing and separating these very water-soluble PFAS. Chromatographic separation approaches divide into applications that rely upon hydrophilic interaction liquid chromatography (HILIC), reversed-phase columns, and ion-exchange columns (Table S8). Instruments used for ultrashort-chain PFAS separation and quantification range from LC–MS/MS to LC-HRMS, as well as supercritical fluid chromatography (SFC) with MS/MS. Given the analytical challenges, the analysis of ultrashort-chain PFAS requires an analytical method separate from those used to concentrate and separate ≥C4 PFAS.

A review by Björnsdotter et al. from 2020 includes methods for ultrashort-chain homologues among the PFCA and PFSA classes.^100^ Initially, analyses of TFA were performed on rain and snow by evaporating aqueous samples (500–1000 mL), derivatizing the residue, followed by separation and detection by gas chromatography–mass spectrometry (GC–MS) (Table S8).^101–106^ This laborious process was replaced with off-line SPE-based sample preparation approaches for sample volumes ranging from 50–500 mL, typically involving WAX,^17,35–37,107,108^ mixed mode ion exchange phases,^106,109,110^ and hydrophilic–lipophilic balance (HLB) SPE sorbents (Table S8).^36,106,111^ Only one report documents an online mixed-mode SPE approach for ultrashort-chain PFAS.^66^ The various SPE-based approaches gave (limits of quantification) LOQs ranging from <1 to 200 ng/L (Table S8). The LOQs for TFA tend to be higher than those for other PFCAs, due to instrumental TFA background levels, which is in contrast to low instrumental background levels for TFMS.^110,112^ Instrumental background is defined as obtaining a signal for an analyte (e.g., TFA) that originates from within the instrument. In some cases, concentrations of TFA could not be reported due to high instrumental background.^113^ Others indicate that the volatility of TFMS limited their ability to report concentrations.^113^ An alternative to previously described methods utilizing SPE but with higher detection limits is the relatively simple direct injection of up to 25 μL aqueous sample with analysis by SFC-MS/MS.^111^ A recent review described a combination of target and suspect screening for ultrashort PFAS, which indicated that C2 and C3 homologues of PFAS classes other than just PFCAs and PFSAs are present in wastewater.^113^

## 7 Volatile Target PFAS Methods

Definitions of volatile and semivolatile organic compounds based on boiling point, vapor pressure, and relative chromatographic retention time are reviewed by Eichler and Little.^114^ ASTM defines semivolatile organic compounds as those with vapor pressures from 10^–2^ to 10^–8^ kPa at 25 °C, while volatile molecules are defined by vapor pressures >10^–2^ kPa at 25 °C.^115^ The World Health Organization (WHO) defines semivolatile compounds as those having boiling points between 240 and 400 °C and vapor pressures of 10^–2^ to 10^–12^ kPa.^116^ Measured and computationally modeled estimates of PFAS vapor pressures are listed on the EPA CompTox Dashboard.^117^ Additionally, vapor pressures have been experimentally determined for nine PFAS.^118^ Eichler and Little used the WHO definition to indicate that homologues within a PFAS class range from volatile to semivolatile, with higher fluorinated chain length homologues falling into the semivolatile category.^114^ It is important to use the vapor pressures of the protonated free acid forms of PFCAs and PFSAs with caution since these neutral forms of PFCAs and PFSAs are not relevant in semiconductor wastewater at circumneutral pH. For wastewater analysis, it is likely that the volatile PFAS listed in EPA Method OTM-50, such as fluoroalkanes and fluorinated greenhouse gases, will partition out of water into the gas phase, thus requiring the need for gas-phase capture and analysis methods that are not covered in this review.

For simplicity and the purposes of this review, the target PFAS listed in Table S9 will be referred to hereafter as “volatile” PFAS. The PFAS listed in Table S9 occur as neutral, nonionized molecules because they either do not have an ionizable functional group (e.g., fluorotelomer acylates and methacrylates) or possess ionizable functional group, such as a hydroxyl group, with a p*K*_a_ that is too high for the molecule to occur in its ionized form in circumneutral pH wastewater (e.g., FTOHs).

### 7.1 Sample Preparation for Volatile PFAS Analysis

#### 7.1.1 Solid Phase Extraction

Methods for volatile PFAS analysis utilize SPE to concentrate volatile PFAS onto SPE sorbents including HLB^119,120^ and WAX (Figure 2 and Table S10).^121^ Volatile PFAS are then eluted with an organic solvent such as methanol or a mixture.^120,121^ The mass of the SPE sorbent and volume of the SPE elution solvent should be taken into consideration because large SPE cartridges require greater volumes of elution solvent. Larger volumes of eluting solvent lowers the concentration of volatile PFAS in the final extract, thus requiring further concentration to maintain sensitivity, ideally using a keeper solvent to prevent loss and maintain sensitivity prior to analyses by GC–MS or LC–MS analysis.^122,123^

**Figure 2:**
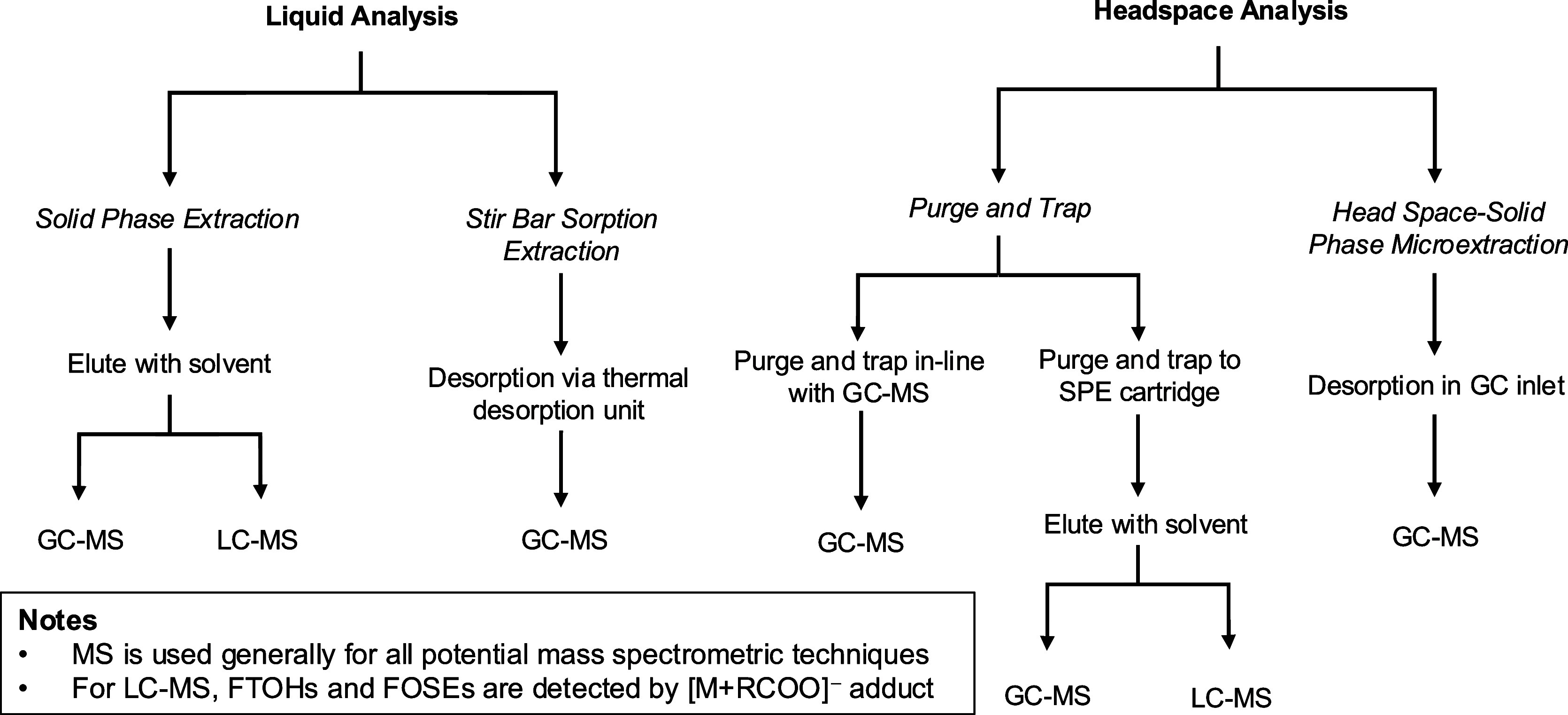
Sample preparation for volatile PFAS in wastewater.

Another method of SPE that interrogates the liquid phase is stir bar sorptive extraction (SBSE) using stir bars coated with polydimethylsiloxane (PDMS; Table S10).^124^ The stir bar is spun in a vial of water, removed, rinsed with deionized water, dried with tissue paper, and desorbed via thermal desorption (TD) into a GC–MS. This method utilizes minimal sample preparation and low method detection limits (≤6.7 ng/L).^124^ While SBSE was successfully demonstrated for FTOH analysis in water,^124^ future work is needed for other classes of volatile PFAS. Additionally, a TD unit is necessary to desorb volatile PFAS from the stir bar, which increases the cost of analysis. Sample preparation for volatile PFAS in water is also performed by methods like those reported for nonvolatile PFAS (Figure 2). For example, liquid–liquid extraction^125,126^ is used to extract volatile PFAS from water.

#### 7.1.2 Headspace Measurements

A common method for the analysis of volatile PFAS in water is to interrogate the gas phase of a sample in a closed system. Published methods for volatile PFAS in water include purge-and-trap^271^ and head space-solid phase microextraction (HS-SPME)^272^ for the determination of volatile PFAS, including FTOHs, FOSAs, fluorotelomer acrylates (FTACs), fluorotelomer methacrylates (FTMACs), perfluoroalkyl iodides (PFAIs), and fluorotelomer iodides (FTIs) (Table S10). In the purge-and-trap method, nitrogen is used to purge volatile PFAS from water onto an activated charcoal filter and subsequently eluted from the filter using a volatile solvent and analyzed via GC–MS.^271^ Other studies describe purging sample headspace onto an HLB^128^ or C18 SPE cartridge.^129^ Once SPE cartridges are eluted with a volatile solvent, extracts are then analyzed via GC–MS or LC–MS. The purge-and-trap method can also be used in line with GC–MS, minimizing the need for solvent elution.

For HS-SPME, a divinylbenzene/carboxen/polydimethylsiloxane (DVB/CAR/PDMS) fiber^130,131^ is typically utilized for extraction from a sample’s headspace in a closed system. The vial, containing a water sample, is agitated via stirring and heated to promote complete volatilization into the headspace. Sodium chloride salt may also be added to promote volatilization through electrostriction of the volatile PFAS. The fiber is exposed to the headspace for 5 to 30 min to reach equilibrium. No solvent is needed for extraction since the fiber is desorbed directly in the inlet of the GC. Recoveries by HS-SPME range from 92–130% for spiked tap water.^130^ The HS-SPME method has a simple sample preparation with high precision while mitigating the potential for contamination. However, when using HS-SPME, other molecules in the sample matrix may compete for adsorption to the fiber, which would result in lowered detection limits. Additionally, carryover occurs if the fiber is not fully desorbed in between sampling, with larger molecules being more strongly retained to the fiber’s coating.^130^

### 7.2 GC–MS and Detectors

Typically, separations of volatile PFAS are conducted via GC. For GC separation, wall-coated open tubular columns are utilized for separation. The most common types of detectors for volatile PFAS analysis by gas chromatography are the electron capture detector (ECD) and MS (Figure 3 and Table S11).

**Figure 3:**
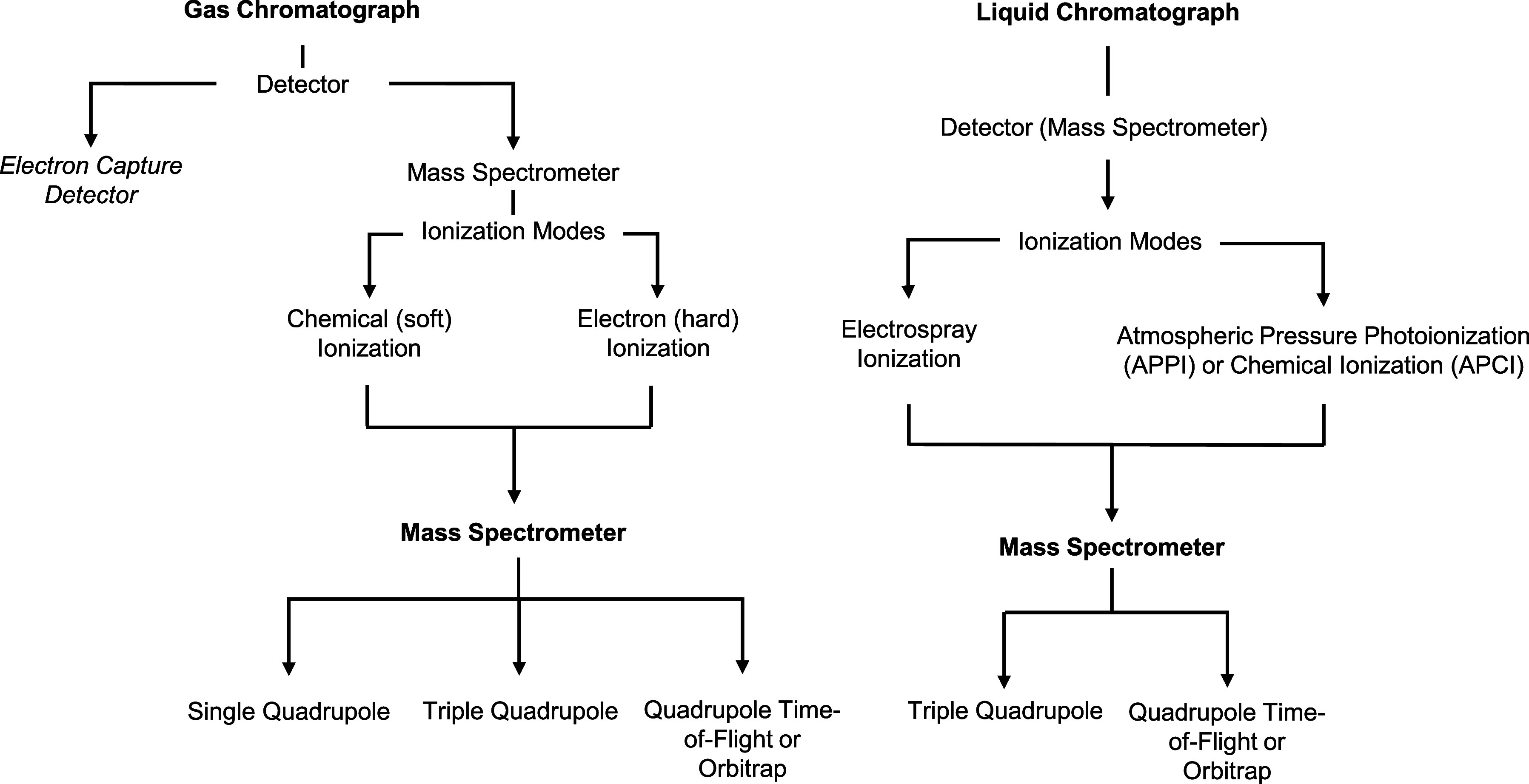
Separation and detection techniques for volatile PFAS in wastewater.

#### 7.2.1 Electron Capture Detector

The ECD is a selective detector commonly used for organo-halogen detection when coupled with GC. For ECD, an electron is released from a radioactive beta-emitter such as ^63^N, which is then attracted to electronegative atoms such as fluorine or chlorine. As the electron is captured, there is a change in current, allowing for halogenated molecule detection. Few studies utilize GC-ECD for volatile PFAS. Historically, GC-ECD was used for the detection of derivatized PFCAs^131,132^ and for monitoring FTOH biodegradation.^133^ Total organic fluorine following PFAS defluorination and derivatization has also been conducted with ECD.^134^ However, ECDs do not offer structural information. Furthermore, comparative studies between GC-ECD and GC-single-quadrupole MS illustrated that LODs (limits of detection) are higher for GC-ECD analysis and therefore less sensitive.^132^ The ECD is also sensitive to all halogens, which may be an issue with the presence of chlorinated, iodinated, or brominated volatile organic compounds.

#### 7.2.2 Single Quadrupole and Triple Quad MS

In contrast to the ECD, single-quadrupole MS is the most utilized detector. Sources of ionization for GC–MS are electron ionization (EI) and chemical ionization (CI) (Figure 3 and Table S11). For volatile PFAS detection, both EI and CI are used for volatile PFAS. For EI, this ionization method is considered a “hard” ionization because the molecule is extensively fragmented with electrons produced by a heated filament, typically at 70 eV. The resulting fragmentation of the molecule allows for structural database matching. However, there exists a potential for artifacts that may confound interpretation of data for PFAS by GC–EI–MS. Roth et al. reported the presence of PFOA in the headspace above water (containing aqueous film-forming foam) at neutral pH.^135^ In response to the work by Roth et al.,^135^ Titaley et al. demonstrated the formation of perfluoro-1-heptene upon injection of PFOA into a 280 °C inlet of a GC–MS, indicating that PFOA cannot be distinguished from the thermal degradation product, perfluoro-1-heptene, using GC–EI–MS.^270^ Others report perfluoroalkenes as thermal degradation products of PFCAs.^136,137^ Hayes et al. later confirmed that PFOA degrades to perfluoroheptene, which gave similar fragments as PFOA (*m*/*z* 69, 119, 131, and 169) under GC–EI–MS conditions.^138^ Thus, a body of evidence indicates that PFOA and perfluoroheptene as well as other analogous PFCAs and their corresponding perfluoroalkenes cannot be differentiated by GC–EI–MS used in soil gas and vapor intrusion studies.^138–140^ They speculated that Roth et al.^135^ detected the aerosolized PFOA in the headspace above circumneutral pH water due to agitation of the solution. Thus, caution is urged when using other analytical techniques that can generate aerosol forms of nonvolatile PFAS, such as purge-and-trap. Furthermore, recent reports of Henry’s law coefficient measurements for *n*:2 FTS^141^ are also based on fragments and not molecular ions resulting in identifying artifacts as FTS.^142^ Thus, without molecular ion confirmation, the data should be treated cautiously.

In contrast, CI is a “soft” ionization that produces less fragmentation and enhances the abundance of the molecular ion, allowing for molecular weight confirmation, which is used to confirm a chemical’s identity. The use of CI also allows for differentiation between individual volatile PFAS based on their molecular weights.^143–145^ For volatile PFAS, it is common to use positive CI (PCI) since the functional group of the PFAS picks up a proton from a reagent gas, typically methane, to form the [M + H]^+^ ion, where M is the unfragmented molecule/analyte. With CI, the molecular ion has a greater intensity and provides an additional line of evidence for chemical identification. Lowering the eV used in EI-MS could be done for molecular ion enhancement as a “pseudo-CI”.^146^ Ayala-Cabrera et al. reported instrumental LODs for fluorotelomer olefins (FTOs), FTOHs, FOSAs, and FOSEs using GC-EI-MS and GC–CI–MS ranging from 0.2–6 μg/L and 0.06–4 μg/L, respectively, with the exception of the 4:2 FTO, which was 17 μg/L and 429 μg/L, respectively.^143^ Thus, there is little difference in instrumental LODs for EI-MS or CI-MS.

Triple quadrupole mass spectrometry (TQ-MS) is coupled with GC for target volatile PFAS analysis. The use of MRM increases both sensitivity and specificity with respect to the single-quadrupole MS. An application note from Shimadzu couples HS-SPME with GC–MS/MS for the analysis of several classes of volatile PFAS. While the instrumental LOD is not reported, the lowest point on the calibration curve ranges from 2.5 to 25 ng/L due to increased sensitivity from MRM.^147^ The lowest calibration point reported here is up to 1000-fold more sensitive than other reported instrumental limits of detection for volatile PFAS using the single-quadrupole MS.^147^

#### 7.2.3 GC-HRMS

Recently, GC-HRMS, using quadrupole time-of-flight (QTOF)^148,149^ and Orbitrap MS,^150^ was used for suspect and nontarget volatile PFAS in matrices of soils,^148,149^ drinking water,^148,149^ and incinerated soils.^150^ Only a small fraction of GC-HRMS databases contain mass spectral fragmentation for PFAS, resulting in a research gap surrounding nontarget volatile PFAS analysis.^150,174^ Casey et al. developed a nontarget work flow for volatile PFAS in incinerated AFFF impacted soils, but the workflow was not applied to water samples.^150^ Additionally, an accurate mass library for volatile PFAS using GC-EI-QTOF was developed by Agilent and applied to drinking water;^149^ however, the accurate mass library only contains EI spectra.^149^ In contrast, GC-PCI-QTOF was used to identify long-chain FTOHs and FTACs, FTMACs, Ft-esters, and FT-ether dimers in industrial soils (but not water) following methyl *t*-butyl ether solid–liquid extraction.^148^ The use of EI is typically favored due to spectral database matching. However, Casey et al. note negative chemical ionization (NCI) needs to be explored because more PFAS were observed in NCI in contrast to PCI or EI.^150^

#### 7.2.4 Artifacts in GC–MS Analysis

Potential artifacts arise in GC–MS analysis due to the presence of nonvolatile PFAS in sample extracts. For example, nonvolatile 6:2 dialkyl fluorotelomer phosphate (diPAP) underwent thermal degradation in the GC inlet at 280 °C to form 6:2 FTOH in a methanol extract. The phenomenon resulted in a false positive for 6:2 FTOH in subsequent injections. Therefore, strong anion exchange SPE was used to remove 6:2 diPAP from extracts.^237^ A cooled GC inlet may also mitigate thermal transformation.^237^ However, as a result of using a low-temperature inlet, nonvolatile PFAS will remain in the GC inlet until it is replaced. Another noted artifact was identified with isotopically labeled FTOH standards. When using PCI, hydrogen abstraction of isotopically labeled FTOH standards yielded false positives for native (nonisotopically labeled) FTOHs, thus requiring blank subtraction or the use of alternative isotopic standards.^151^ Further research on other PFAS thermal transformations in the GC is needed to understand potential artifacts that may arise.

### 7.3 LC–MS for Volatile PFAS

#### 7.3.1 LC Electrospray Ionization

While GC–MS is historically used for the determination of volatile PFAS, LC–MS/MS methods list several classes of volatile PFAS, such as FTOHs,^120,121,126,152,153^ FASAs,^154^ and FOSEs^155^ (Figure 3 and Table S9). There are no standardized (e.g., EPA) methods for volatile PFAS analysis in water, with the exception of EPA Method 1633 that targets two FOSEs, namely, Me- and Et-FOSE.^25^ Using ESI, FASAs, specifically Me- and Et-FOSA, are readily ionized to form the [M – H]^−^ ion, but other classes of volatile PFAS are poorly ionized, resulting in a high LOD. In buffered aqueous mobile phases, FTOHs and FOSEs (Table S9) are detected in negative mode as acetate adducts when undergoing ionization by ESI.^154,156^ Alternatively, FTOHs are observed as their [M – H]^−^ ion in ESI^–^ but only with unbuffered mobile phases. However, without buffered mobile phases, nonvolatile PFAS give higher detection limits, which complicates attempts to simultaneously analyze nonvolatile PFAS and volatile FTOHs.^152^

Berger et al. compared the use of an ion trap MS, TOF MS, and TQ-MS and reported LODs as a function of mass on column.^152^ The ion trap MS had LODs ranging from 1 to 10 ng on column for the 4:2, 6:2, and 8:2 FTOH. In contrast, the TOF MS had LODs ranging from 0.005 to 0.15 ng on column, and the TQ-MS had LODs ranging from 0.001 to 0.02 ng on column.^152^ Therefore, the LC-TQ-MS is the most sensitive for the quantification of FTOHs with LC. The LC-TQ-MS LOD is an order of magnitude higher than reported instrumental LODs for GC-TQ-MS (0.0001–0.002 ng mass on column).^157^ Thus, GC–MS tends to be more common for volatile PFAS analysis. Additionally, most volatile PFAS analysis by LC–MS/MS has focused on FTOHs, FOSAs, and FOSEs. Further research is needed on other classes of volatile PFAS, ionization efficiency, and detection with ESI.

#### 7.3.2 Atmospheric Pressure Photoionization and Atmospheric Pressure Chemical Ionization

While most volatile PFAS LC–MS analyses are conducted in ESI negative mode, a few studies have also compared different modes of ionization for volatile PFAS when using LC–MS (Table S11).^120,143,158^ The most comprehensive was described by Ayala-Cabrera et al. in which they compared different ionization methods for FTOHs, FOSAs, FOSEs, and FTOs using APCI and atmospheric pressure photoionization (APPI) with tandem MS.^143^ To the best of our knowledge, this was the first study to identify four classes of volatile PFAS targets simultaneously with alternative interfaces in combination with MS/MS detection. In the same study, LC-APCI-MS/MS and LC-APPI-MS/MS gave instrumental LODs ranging from 0.3 to 100 μg/L and 0.1 to 4000 μg/L, respectively.^143^ The LODs were higher for LC-APPI-MS/MS and had LOD ranges larger than those of GC–MS methods. However, LC-APCI-MS/MS yielded less than 1 μg/L LODs for the volatile PFAS studied except for the 4:2 and 6:2 FTO.^143^

#### 7.3.3 Derivatization of FTOHs for LC–MS/HRMS

To overcome the poor ionization and high detection limits of FTOHs via LC–MS/MS analysis, Peng et al. demonstrated the use of derivatization for FTOH. Derivatization is the process by which an analyte is chemically altered to improve chromatographic separation and/or detection. Derivatization was performed using dansyl chloride with a 4-(dimethylamino)-pyridine catalyst so that FTOHs could be analyzed via LC–MS/MS.^159^ This method yielded lower LC–MS/MS detection limits by 7.5–841-fold when compared to nonderivatized FTOH analysis and 57–357-fold lower detection limits for FTOHs when compared to a GC-MS method.^159^ This derivatization method was applied to analysis of FTOHs in sediment,^159^ wastewater,^160^ and food packaging.^161^ While derivatization is useful for FTOH analysis, FTOHs constitute one class of volatile PFAS. Thus, derivatization of other volatile PFAS needs to be explored for improving detection via LC–MS. Furthermore, it is unknown how this derivatization reaction may impact other PFAS. Additionally, derivatization is time-consuming and requires multiple transfer steps that could lead to loss and reduced precision.

#### 7.3.4 Implications for Semiconductor Wastewater

Volatile PFAS were measured in wastewater of fluorochemical-related facilities such as a durable water repellent facility^119^ and a textile manufacturer,^162^ and volatile PFAS were detected in air surrounding wastewater.^163^ Ma et al. determined FTOHs in municipal wastewater,^162^ and Mok et al. determined FTOHs, FTACs, and fluorotelomer acetates (FTATs) in wastewater from fluorochemical-related facilities, using targeted and nontargeted GC–MS approaches.^119^

Neutral PFAS noted in orange in Tables S4 and S5 used by the semiconductor industry are not ionized in the environmentally relevant range of pH values, nor under electrospray (LC-HRMS) conditions. Therefore, extraction methods for volatile PFAS coupled with GC-HRMS are needed for nontarget analysis of neutral PFAS since their ionization under LC-HRMS conditions is unknown. While the molecules shown in Tables S4 and S5 are potentially used by the semiconductor industry, their occurrence and fate in semiconductor wastewater is currently poorly understood. Additionally, the identification of volatile PFAS is necessary for calculating total fluorine mass balances in semiconductor wastewater.

## 8 Suspect and Nontarget PFAS Workflows for High-Resolution Mass Spectrometry

It is challenging to know all of the potential PFAS present in semiconductor wastewater given the number and complexity of semiconductor fabrication units. Nondisclosure agreements also limit the information available to the public on the PFAS used in semiconductor processes. Furthermore, PFAS used in semiconductor processes may react to generate novel, unknown transformation products. Additionally, PFAS intentionally added to formulations used during semiconductor processes may contain up to 0.5% byproducts.^16^ Finally, intermediate transformation products are formed during biological oxidation in wastewater treatment plants receiving semiconductor waste.^17^ Impurities and transformation products are generally not captured by analytical methods focusing on target PFAS, such that nontarget workflows must be used to account for these potential PFAS.

Several PFAS found in semiconductor wastewater were discovered using nontarget workflows (Tables S2 and S3).^16,17,19^ In addition, there are PFAS potentially associated with the semiconductor industry (Tables S4–S6), for which there are no wastewater data. Some of these novel PFAS are listed in suspect screening lists, where suspect PFAS are defined as previously identified PFAS but have no commercial analytical standard.^164,165^ Until analytical standards become available for all relevant PFAS present in semiconductor wastewater, investigations will require suspect and nontarget PFAS analyses that require sophisticated identification and data techniques. The general concept of suspect and nontarget analysis is to gain enough structural information on an unknown PFAS from HRMS data to increase the confidence in the identity of the PFAS to the point that a probable structure can be communicated with an established level of confidence.^166,167^

Sample preparation and chromatographic separation constrain the PFAS that can be detected and ultimately identified.^168^ As discussed, nonvolatile and volatile PFAS require different approaches to sample preparation and separation methods prior to detection.^169^ One limitation of MS is the prerequisite that PFAS must be ionizable. A recent model offers guidance on how to determine if a chemical is amenable to LC–MS analysis.^170^ Using MS, PFAS is detected as either negatively or positively charged molecules. Most target PFAS (Tables S1 and S9) are negatively charged and are thus detected in negative mode only. Consequently, PFAS detected in positive mode (e.g., zwitterionic and cationic), if present in semiconductor wastewater, would go undetected if only negative-mode detection is employed.^171^ To the best of our knowledge, only Jacob et al. looked for PFAS in semiconductor wastewater under positive mode but found none.^19^

High-resolution mass spectrometers come in many different forms, including those interfaced with LC or GC instruments. Because benchtop GC-HRMS have become only available recently,^150,163^ few nontarget PFAS studies have used GC-HRMS.^150,163,172^ Prediction models estimate that only 10% of PFAS are amenable to LC-HRMS analysis.^173^ Therefore, there is potential for determining nontarget PFAS using GC-HRMS. Strategies described in this section are similar, to some extent, to those for LC-HRMS. The data collected include retention time, peak shape, accurate mass, and molecular fragments (MS2) for each detected peak (Table S12). The certainty with which a molecular fragment can be identified is associated with the resolution and mass accuracy determined by the type of HRMS instrument used to acquire the data.^174^ Resolution is the ability of an instrument to distinguish two peaks with slightly different masses, whereas mass accuracy defines how accurately the mass is measured. Two of the most common types of HRMS instruments found in laboratories are the QTOF and Orbitrap mass spectrometers. The QTOF mass spectrometer has a resolution up to 60,000 (unitless), a mass accuracy lower than 10 ppm, and an upper mass cutoff of ∼*m*/*z* 2000. In contrast, the Orbitrap mass spectrometer (ThermoFisher Scientific) has a resolution up to 500,000 (unitless), a mass accuracy of 1 ppm, and an upper mass cutoff of *m*/*z* 8000. Additionally, scan speed varies between instruments where a higher scan speed allows for more MS2 data collection, thereby increasing the number of potential PFAS identified. Most QTOF instrument scan speeds are generally <0.05 s, while an Orbitrap’s instrument scan speed is ∼1 s.^167^ Another type of HRMS is Fourier-transform ion cyclotron resonance MS (FT-ICR-MS),^175^ which offers the advantage of ultrahigh-resolution up to 1,000,000 with a mass accuracy below 1 ppm. Limitations of FT-ICR MS include the cost of operating the instrument, no chromatographic separation due to direct injection, as well as complex data processing workflows.^175^ Finally, ion-mobility MS^176^ adds collision cross-section as a parameter, which improves confidence in the identity of unknown PFAS.^177,178^ Collision cross-section for PFAS has been recently aggregated into PubChem and open-source NTA database to support this effort.^179,180^

### 8.1 Workflows

To date, QTOF and Orbitrap HRMS are the most common instruments for suspect and nontarget PFAS studies. Both QTOFs and Orbitraps are becoming more cost efficient, such that they are now used by contract laboratories. There are excellent reviews that give an overview of nontarget analysis and best practices,^167,181^ as well as practical reviews on discovering novel PFAS.^171,182^ Since such reviews exist, this section focuses on a practical overview of workflows used to find novel PFAS in semiconductor industry wastewater.

Suspect and nontarget PFAS identification using HRMS data features are performed using workflows (Table S13), which are multistep methods meant to elucidate the identity of unknown chemicals. Prioritization of masses of interest is made by matching the mass of peaks to those on suspect screening lists.^164,165,183,184^ An accurate mass corresponding to a PFAS is selected by mass-defect filtering^185^ along with Kendrick mass defect (KMD) analysis to find PFAS that occur as a homologous series.^19,47,186^ Mass fragment patterns are matched to those in suspect PFAS databases.^187^ In case of nontarget PFAS, characteristic mass fragments and differences between mass fragments are used to identify or suggest potential PFAS structures.^17,47,98,188^ This last step is assisted using computer-based methods (e.g., in silico, MS2 reconstruction).^189^ The retention time of an unknown PFAS also provides an additional line of evidence to assist in assigning confidence in the identity of the unknown PFAS. For example, the retention time of a higher-molecular weight homologue within the same PFAS class should be at a greater retention time than those of lower-molecular weight homologues.^190^ Several vendor or open-source software tools generally contain a combination of methods, many of which were previously compared.^19,191–195^ All workflows contain pros and cons that should be evaluated to meet specific goals. The capability to investigate the structure is mainly dependent on the MS2 fragmentation quality, which depends on the type of HRMS acquisition (Table S12).

#### 8.1.1 Semiquantification

Calibration curves cannot be made for suspect and nontarget quantification since they do not have analytical standards. Thus, diverse strategies have been developed and applied to give estimated concentrations of suspect and nontarget PFAS.^17,59,188,196–198^ Despite the lack of best practices, the most common semiquantitative method is to use the “best matched” approach. Using the “best matched” approach, a suspect or nontarget PFAS is assumed to have the same response factor as that of a target PFAS that most closely matches the suspect or nontarget PFAS molecular structure or chromatographic retention time.^17,59,188^ However, the response factor is a function of the ionization efficiency, which varies depending on the PFAS structure (e.g., number of fluorine atoms as well as the headgroup).^196^ Moreover, selecting the “best match” is subjective and thus may result in variability in estimated suspect and nontarget PFAS concentrations. A more complex estimation method was recently described^197^ but has some practical limitations. On the other hand, Cao et al. proposed a simpler approach using “an average calibration curve” based on all target PFAS that was used to estimate suspect PFAS concentrations.^198^ This method is very practical and easy to implement and has the advantage that the uncertainty about the estimated concentration is calculated.

## 9 Nonspecific Methods for PFAS Analysis

Total fluorine is defined as the combination of inorganic forms of fluorine (e.g., fluoride ion and inorganic anionic fluorine salts) and organic fluorine.^23,134^ Nonspecific methods are a collection of methods aimed at capturing organic fluorine by incorporating steps to remove forms of inorganic fluorine^134^ and are valuable as targeted techniques account for less than 10% of total organic fluorine detected in samples.^23,199,200^ Nonspecific analytical methods could benefit the semiconductor industry as target PFAS are a fraction of the PFAS in semiconductor wastewater.^16,17,19^ Nontarget screening approaches reveal unidentified PFAS that contribute to total organic fluorine.^16,17,19,201^ At present, there is only one EPA-approved nonspecific PFAS method (EPA Method 1621) for adsorbable organic fluorine.^202^ For the purposes of this review, nonspecific methods are divided into those that are generally practiced with sample preconcentration, and those that are not (Figure 4 and Table S14).

**Figure 4:**
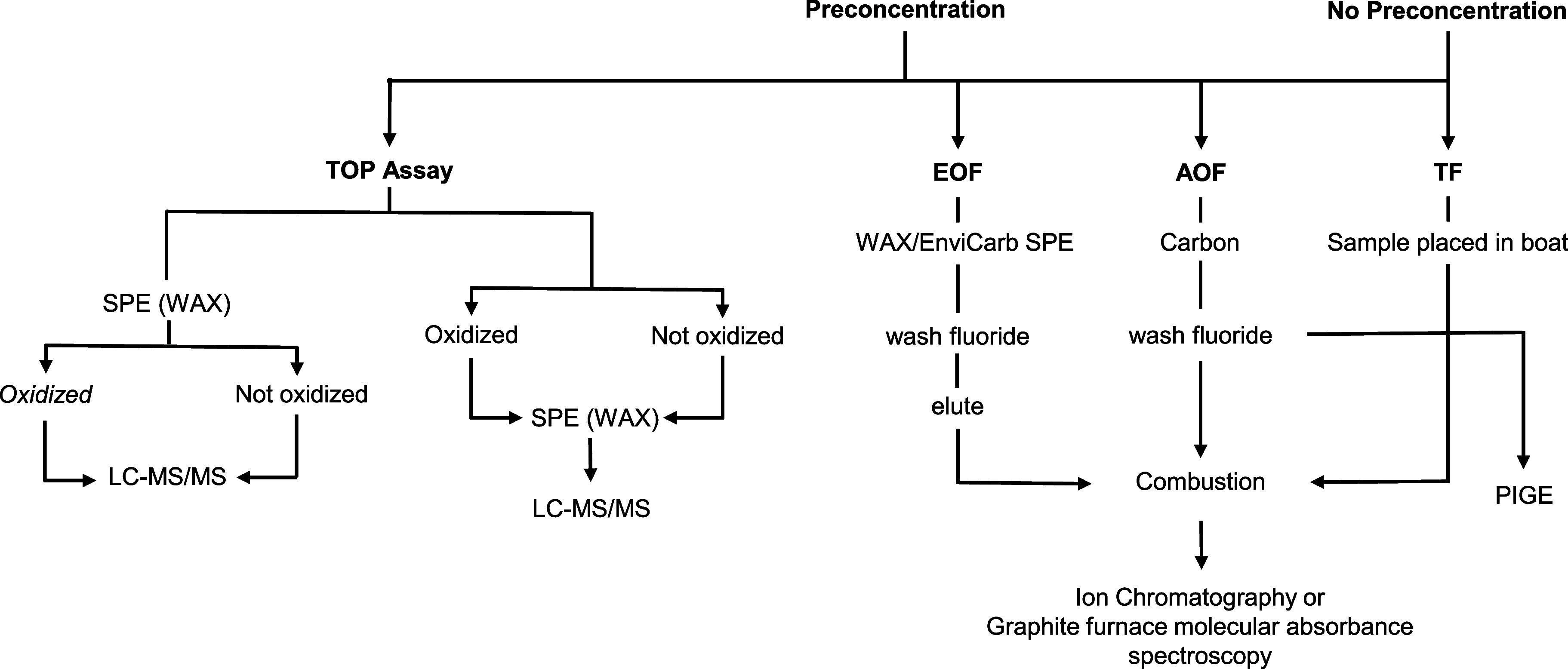
Sample preparation and instrumental analysis approaches for nonspecific methods of PFAS detection.

### 9.1 Total Oxidizable Precursor Assay

The total oxidizable precursor (TOP) assay provides a quantitative estimate of precursors that form target PFCA and PFSA upon oxidation.^203,204^ The TOP assay is the only nonspecific method that relies on the measurement of target PFAS (i.e., PFCAs). For the TOP assay, there are two approaches. In the first approach, the water sample is preconcentrated by WAX SPE after which the extract is split and one aliquot is treated by heat- and alkaline-activated persulfate oxidation, while the other aliquot is left untreated (Figure 4).^49,205,206^ In the second approach, the sample is split first and one fraction is oxidized while the other fraction is not, then the two fractions are preconcentrated by WAX SPE (Figure 4).^207–210^ Precursors are then quantified as the net production of PFAAs after oxidation. Note that concentrations are often reported as the summed concentration of ng/L for convenience rather than in molar concentration units. To date, there is a single report of PFSA and PFASAs as minor oxidation products from the oxidation of an electrofluorination (ECF)-derived zwitterion precursor.^206^ There is one study highlighting mixed-mode SPE to evaluate the cationic-, zwitterionic-, and anionic-precursor fractions in AFFF impacted sites.^211^ This study evaluated groundwater, untreated influent, and treated effluent from wastewater plants, signifying potential value to the semiconductor industry in utilizing the methods outlined elsewhere.^211^

The TOP assay has low detection limits compared to other indirect methods for PFAS (Table S14) and is attributed to the low instrumental LC–MS/MS detection limits as well as the preconcentration steps.

Originally, the TOP assay measured PFCAs ≥C4 (e.g., PFBA) due to the availability of analytical standards. Most sample preparation and chromatographic separation methods (e.g., LC–MS/MS) are therefore optimized for PFBA and PFCAs of longer chain lengths. Recently, ultrashort-chain PFCAs including TFA^38,212^ and PFPrA were included.^49^ Thus, omitting ultrashort-chain PFCAs from the TOP assay is recognized as a potential source of low bias in estimated precursor concentrations since ultrashort chains are common products of oxidation.^24^ Patch et al. determined that maintaining the postoxidation sample above a pH of 7 resulted in limited conversion of PFCAs to shorter chain products; such that with thermal activation alone, it is possible to limit conversion of precursors into ultrashort-chain analytes.^213^ Patch et al. also determined that UV radiation at 254 nm activated the oxidant more rapidly than solely thermal activation, resulting in 93% activation within the first hour, resulting in partial conversion of PFOA into PFHpA and PFHxA, while 6:2 FTS underwent rapid transformations both in thermal and UV-activated experiments.^213^ Further, Patch et al. determined that, in their UV experiments, the least loss of PFOS resulted from quenching the oxidation reaction immediately with methanolic acetic acid and then diluting to subsample and run the extracts on the instrument.^213^ Tsou et al. recommend monitoring the oxidation of a stable isotope precursor (e.g., ^13^C_8_ FOSA) compared to the labeled product (^13^C_8_ PFOA)^212^ or monitoring for oxidizable precursors as target PFAS alongside PFCAs.^24^

Recent investigations have characterized the reactivity of a more diverse array of PFAS under TOP assay conditions. Structures that are resistant to oxidation include perfluorinated ethers,^38,210,214^ unsaturated PFSAs,^214^ and bistriflimide (Table S6).^215^ In contrast, polyfluoroalkyl ether acids with an –O–CFH– moiety were readily oxidized into ultrashort-chain PFAS.^210^ The perfluorinated alcohol, hexafluoropropanol, gave 17% molar conversion to TFA.^215^ In studies looking at environmental water impacted by AFFF, cationic precursor PFAS yielded 74–103% recovery, while zwitterions and neutral recovery were as low as 28% recovery.^211^ This signifies that more PFAS can be selectively quantified using mixed mode SPE, which could be of potential value to the semiconductor industry.^211^

Further investigations to improve the TOP assay include removal of dissolved organic matter, which is shown to interfere with the TOP assay.^216^ Patch et al. determined that an application of 500 mM hydrogen peroxide for 22 h gave the highest yield of PFCA in the presence of up to 1000 mg/L total organic carbon.^217^ However, relevance to the semiconductor industry may be limited, as the effluent is typically not considered high in organic matter.

The TOP assay preserves some information about the nature of the fluorinated chain of precursors, including both chain length and branching. The fluorinated chain length of ECF-based precursors is preserved in the PFCAs that result from oxidation.^38,49,212^ For example, many C8 ECF-based precursors yielded primarily PFOA as a product.^49^ However, chain length information is not conserved for fluorotelomer-based PFAS, since oxidation of these PFAS generates an array of shorter-chain PFCAs.^49,212^ For fluorotelomer precursors, the longest chain length PFCA generated carries the same number of fluorinated carbons as the precursor as well as an additional carbon in the carboxylic acid functional group. For example, 4:2 FTS yields the C5 PFCA (PFPeA) as the highest chain length PFCA after oxidation.^49^ While such interpretation is possible with individual precursor standards, interpreting the PFCA distribution from environmental samples is more challenging. However, Antell et al. reported that incorporating the TOP assay PFCA distributions gave improved separation of PFAS source waters (e.g., wastewater effluent and landfill leachate) by principal component analysis.^218^

### 9.2 Extractable Organic Fluorine and Adsorbable Organic Fluorine

At the time of this review, several comprehensive reviews exist that provide the history and detailed discussions on extractable organic fluorine (EOF) and adsorbable organic fluorine (AOF)^134^.^23^ Both reviews document several alternatives for sample preparation and fluorine detection.^23,134^ There are numerous reports that compare the performance of EOF and AOF for individual target PFAS as well as for environmental samples.^219–221^ Given the extensive literature on this topic, only the most common are discussed in this review with a focus on those most potentially applicable to semiconductor wastewater.

One distinction between the EOF and AOF is the approach to sample preparation. To measure the EOF, samples are concentrated using SPE sorbents, while carbon-based sorbents are used in AOF (Figure 4). Another distinction is that EOF sorbents are eluted to create an extract that undergoes combustion, while in AOF, the carbon sorbent is directly combusted. Both EOF and AOF utilize combustion ion chromatography (CIC) to pyrolyze fluorine-containing compounds at 900–1000 °C in an oxygen-rich environment into fluoride ions that are quantified by ion chromatography.^219,222^

### 9.3 Extractable Organic Fluorine

Given the premise of the EOF, selection of the SPE sorbent is critical. The original as well as more recent EOF methods utilize WAX SPE, thus focused on the concentration of anionic PFAS.^107,221,223,224^ Alternative SPE sorbents used for EOF include combinations of WAX and graphitized carbon black (GCB)^219^ and HLB.^220^ The LOD of EOF methods depends on a number of sample preparation factors but ranges from 0.05 to 11 μg/L fluoride.^23^

The effect of chain length and EOF recovery was documented with recoveries ≥70% for ≥C3 PFCAs^219^ and ≥C4 PFSAs.^23,223^ However, ultrashort-chain PFAS, e.g., TFA and TFMS, gave recoveries of 21% and 52%, respectively. The low observed recovery was attributed to incomplete elution, loss during the SPE drying step, and loss from the washing step to remove fluoride.^221^ A number of per- and polyfluoroether-based carboxylates and sulfonates gave good recoveries by EOF on a WAX/GCB sorbent.^219^ Neutral PFAS (FTOHs) gave lower recoveries (44–55%) while zwitterionic PFAS gave comparable but lower recoveries than equivalent chain-length PFCAs or PFSAs.^219^ The recovery of weak acids, such as the FASAs and their Me- and Et-substituted forms, from water at pH less than five by WAX SPE has not been investigated. While weak acids may sorb more onto a mix of WAX/GCB sorbent, loss of volatile PFAS may occur during the SPE drying step and reduce the final calculated concentrations of PFAS.

After evaluating the roles that the pH of water sample, sample volume, SPE wash step, SPE drying time, and elution strategies have on PFAS recovery, Forster et al. recommend adjusting the pH to less than five with nitric acid and washing with 0.1% NH_4_OH.^219^ The performance of a WAX-based EOF method was sensitive to other parameters, including initial PFAS concentrations, WAX drying times, and CIC parameters.^221^ The use of 0.1% NH_4_OH is a compromise between fluoride removal and retention of short-chain PFAS.^221^ Because of the significant inorganic fluoride present in semiconductor effluent, EOF methods may need further optimization to exclude fluoride while retaining short-chain PFAS. Dixit et al. state that background measurements of materials should be included, as well as potentially utilizing larger sample volumes or different carbon felt sorbents for lower detection limits.^225^ The limits of detection for EOF vary greatly depending on the extraction steps as well as the analytes (Table S14). Typically, ultrashort chain PFAS perform poorly compared to longer-chain PFAS, and the performance of polyfluoroethers has not been evaluated in EOF methods.

### 9.4 Adsorbable Organic Fluorine

Adsorbable organic fluorine methods refer to those methods that rely upon PFAS sorption to carbon-based sorbents that are directly combusted to produce hydrogen fluoride (HF) that is then quantified by ion chromatography (Figure 4).^23,226^ US EPA Method 1621 is an AOF method in which 100 mL of a water sample (pH >5) is mixed with 2 M sodium nitrate (NaNO_3_) and then passed through two 4 mg carbon cartridges. The cartridges are washed with 0.01 M NaNO_3_ and combusted at 1000 °C.^202^ The combustion-generated HF is then analyzed for fluoride by ion chromatography, with a reported detection limit of 2.4 μg/L.^202^ Studies that optimize AOF and compare the concentrations to EOF are now available.^219,221^

Recoveries of ≥C4 PFCAs and PFSAs by AOF methods are most commonly reported and are typically >80%.^23,219,221,222,226^ However, recoveries of PFBA vary from ∼80%^219^ to 70%^221^ but are reported as low as 50%^222^ and 40%.^226^ For the short-chain C3 PFCA (PFPrA), recovery from river water samples at pH <1 was 80%,^219^ but <30% for samples at pH 5 that was attributed to the wash step and losses during the evaporation,^221^ while no recovery was reported from samples at pH <2.^222^ The lack of recovery of C3 PFCA and TFA from pH <2 sample may be due to the activated carbon pretreatment with 0.2 M NaNO_3_ or the washing step with 0.01 M NaNO_3_ ^222^ since sorption from water samples pH <2 is demonstrated by Forster et al.^219^ Recovery of PFPrS at pH 7 was >80%, whereas the recoveries for TFMS and TFA were <20% and attributed to loss during the wash step.^221^ Short-chain ethers (and PFBA) also gave low recovery from AOF when water samples were amended with 0.01 M KNO_3_ (potassium nitrate) and carbon pretreatment and rinses with 0.05 M KNO_3_.^226^

Forster et al. attributed higher recovery of the C3 PFCA to the low sample pH <1, which also minimizes fluoride retention by activated carbon and to the use of 0.01 M NH_4_OH (ammonium hydroxide) instead of KNO_3_ as a wash step.^219^ For example, at low pH, granular activated carbon becomes cationic, potentially sorbing anionic PFAS to a greater degree. However, acidification leads to retention of HF (p*K*_a_ = 3.1)^219^ and may protonate weak-acid PFAS (e.g., FASAs and fluorotelomer carboxylates).^23^ For example, von Abercron reported <30% recovery of the C4 and C6 FASAs when water samples were pH <2.^222^ Recovery of neutral PFAS (FTOHs) were 40–55%, while zwitterionic PFAS were similar to that of PFCAs and PFSAs of similar carbon-chain length.^219,222^ Forster et al. also reported a reduction in PFAS recovery with higher dissolved organic carbon (DOC) levels; therefore, recommending dilution of samples >5 mg/L DOC.^219^

The major factors that influence the performance of AOF methods, especially for short-chain PFAS, include the type of carbon sorbent used and the wash step to remove fluoride.^23,221^ Potential loss of long-chain PFAS during filtration or upon transferring a subsample from original sample bottles for AOF analyses is also described as an area of potential concern.^23^ Incomplete combustion efficiency of PFSAs was indicated as an area that merits optimization by Pan and Helbling.^221^ Limits of detection for AOF, when used in conjunction with CIC, are typically higher than those of other techniques (Table S14). However, when particle induced gamma ray emission (PIGE) spectroscopy is used as the detector, the detection limit is comparable to those of the TOP and EOF (Table S14). However, PIGE cannot distinguish between fluoride and organic fluorine.

### 9.5 Alternative Detectors for AOF

Alternatives to CIC include graphite furnace molecular adsorption spectrometry (GF-MAS), which is based on the formation and detection of GaF (gadolinium fluoride).^220^ Despite its better sensitivity, the behavior of PFAS using this approach is not as thoroughly investigated as for CIC.^23^ Gehrenkemper et al. note that GF-MAS was a more sensitive detector but gave comparable concentrations of total fluorine to CIC.^220^

Particle-induced gamma emission (PIGE), another alternative for quantifying total fluorine, is a nondestructive, high throughput, surface technique (maximum depth of proton penetration is 220 μm) that involves the excitation of atomic nuclei via proton bombardment.^134,227^ Concentrations of total fluorine are determined by comparing counts for the sorbent surface to that of calibration standards, either PFAS standards or sodium fluoride.^203,227,228^ Because PIGE detects both organic fluorine and inorganic fluoride, sample preparation and removal of inorganic fluoride is required.^134,229^ There is one report of PIGE as an alternative detector for AOF where Tighe et al. passed a 2 L water sample through an activated carbon felt sorbent (Figure 4).^228^ The sorbent was removed, dried, and subjected to PIGE analysis. They report fluoride removal for water samples pH ≤2, with a detection limit of 50 ng/L fluoride.^228^ In the study by Dixit et al., AOF quantified by PIGE gave lower detection limits than with CIC analysis, such that PIGE was determined to be a more sensitive detector.^225^

Instrumental neutron activation analysis (INAA) is another alternative for total fluorine analysis. To utilize INAA for water samples, sample preconcentration onto a solid sorbent is required. Total fluorine by INAA is accomplished by bombardment of the solid with neutrons, producing radioactive isotopes, which are then measured.^134,230,231^ Quantification is performed by external calibration.^231^ The advantage of INAA over PIGE is that INAA interrogates the entire solid sample, not just the surface.^231^ To the best of our knowledge, INAA has not been utilized as a detector as part of the AOF methods. However, aluminum can interfere with the detection of fluoride, such that samples with high aluminum content are not suitable for this technique.^230^ Historically, aluminum has been used to remove excess fluoride from effluent in the semiconductor industry.^232^ As a result, there is a potential to have high aluminum levels, thus making INAA unsuitable for semiconductor samples.^232^

### 9.6 Total Fluorine

Total fluorine (TF) is a measure of all fluorine (inorganic and organic) concentrations in a sample. While simple, the method has higher detection limits and captures any inorganic fluoride since there is no sample preparation.^221^ For this reason, TF limits of detection are significantly higher compared with any other indirect methods (Table S14). TF is used to estimate total organic fluorine by subtracting fluoride ion concentrations.^134,201^ If present but not accounted for, any other anionic fluoride forms would contribute to the organic fluorine fraction.^107,134,233^ Shelor et al. note that if background fluoride ion concentrations are high relative to organic fluorine concentrations and comprise nearly 100% of TF, the attempt to quantify total organic fluorine may not be applicable.^23^ TF measurements were performed on semiconductor materials (photoresists and top antireflective coatings ) by placing the materials in boats and combusting the material in an oxygen/argon atmosphere.^201^ The HF produced was trapped in water, and the fluoride was quantified by ion chromatography. Semiconductor wastewater was also directly combusted (no sample preparation) for TF.^201^ TF measurements are then compared against separate fluoride ion concentrations, measures of target and suspect PFAS, and other measures of organic fluorine (see below) to compute and understand mass balances. For example, the inability to close the mass balance on TF was attributed to the presence of nonextractable or adsorbable fluoropolymers.

### 9.7 Implications for Semiconductor Wastewater

The shift to short-chain PFAS by the semiconductor industry indicates that monitoring only for the ≥C4 PFCAs or greater may miss many C1–C3 short-chain precursors, unless methods for ultrashort-chain PFCAs are utilized. To better characterize the PFAS composition of semiconductor materials and wastewater, there may be PFAS that do not oxidize during the TOP assay. For example, the TOP assay did not account for a significant fraction of TF by AOF, which indicates that PFAS in semiconductor wastewater are not oxidizable.^201^ The perfluoro mono- and polyfluoroether acids reported in semiconductor wastewater (Table S3)^19^ are among the classes that do not oxidize in the TOP assay.^210^ Zhang et al. report that resistance to oxidation in the TOP assay corresponds with stability during oxidation-based wastewater treatment.^210^ Given that PFAS are selected for use by the semiconductor industry due to their chemical stability under a range of conditions, it is not surprising that PFAS in semiconductor wastewater may go unobserved by the TOP assay. Thus, caution is urged when using the TOP assay for semiconductor wastewater characterization since it may yield a significant underestimation of precursor concentrations.^219^

In the case of nonspecific methods for organic fluorine, most rely on the quantification of fluoride as a measure of organic fluorine. However, extensive use of hydrofluoric acid^232^ creates significant levels of background in organic fluoride in semiconductor wastewater. Reported fluoride concentrations in semiconductor wastewater range from 500 to 2000 mg/L.^234,235^ The removal of 99% of fluoride could result in concentrations several orders of magnitude greater than measured PFAS concentrations in semiconductor wastewater (10–10,000 ng/L).^17,19^ Therefore, the suitability of nonspecific methods that rely on fluoride detection will depend on extremely efficient fluoride removal steps to ensure that organic fluorine concentrations are not overestimated.

## 10 ^19^F NMR for Quantitative and Qualitative PFAS Analysis

Multiple recent studies highlight the utility of solution state ^19^F NMR spectroscopy for characterizing and quantifying PFAS,^236–239^ identifying PFAS in complex mixtures (e.g., aqueous film forming foams),^240^ and characterizing reactions such as PFAS degradation and PFOS synthesis.^241,242^ The applicability to environmental samples by ^19^F NMR was demonstrated through the analysis of PFAS in rainwater and in consumer products.^236,243^ Advanced ^19^F NMR techniques have been used to study the structure and physical properties of fluoropolymers in solutions.^244–246^

Advantages of ^19^F NMR over other commonly used PFAS characterization techniques include minimal sample preparation, ability to identify and discriminate non-PFAS fluorine (such as inorganic fluoride ion) from organic forms of fluorine, minimal matrix interferences, and its nondestructive nature. NMR tolerates a wide range of sample conditions, being amenable to both organic and aqueous solvents and a relatively broad range of pH values. Like all techniques, NMR has disadvantages, including relatively low sensitivity, susceptibility to interference from paramagnetic ions, and sensitivity to high salt concentrations (>150 mM sodium chloride (NaCl)). While NMR can detect fluorinated compounds, there is no standardized U.S. EPA or ASTM NMR method that has currently accepted NMR-based methods for wastewater analysis.

The basic NMR experiment is relatively straightforward. A lock solvent, such as deuterated water (D_2_O) or methanol (CD_3_OD), and an internal reference standard with a known chemical shift and concentration are added to a sample or extract, transferred to an NMR tube, and loaded into the NMR spectrometer. The sample is then subjected to one or more radiofrequency pulses, and the resulting signal is recorded. The sample remains in the NMR tube for the entire analysis, so there is minimal chance of carryover between samples or contamination during analysis, unless NMR tubes are reused. The sample is not consumed during the analysis, so subsequent analysis using a different NMR experiment or an orthogonal analytical technique (such as LC–MS) is possible. A single internal reference standard, such as hexafluorobenzene^239^ or trifluoroethanol, is usually sufficient for chemical shift referencing and quantification.^247^ In cases where the reference standard cannot be added to the sample, a coaxial tube (a thinner tube that is placed inside the regular NMR tube) is used to keep the sample isolated from the internal reference standard. Chemical shift, δ, is defined as $\delta =\frac{\nu -\nu_{ref}}{\nu_{ref}}\times 10^{6}\;ppm$ , where ν is frequency, and ν_ref_ is the frequency of the internal reference standard. Thus, the chemical shift is normalized by ν_ref_, which makes chemical shifts comparable across magnetic field strengths. It is important to note that chromatographic separation is not required for NMR analysis, which simplifies the analysis workflow while retaining the ability to identify individual compounds in complex mixtures.^248^ In cases where resonance overlap is too severe, a chromatographic step could be incorporated to separate the components of the mixture, or multidimensional (2D or higher) NMR experiments are employed.^249^ NMR is also an invaluable tool for structure characterization and determination. Numerous 1D and 2D NMR experiments exist and are commonly used to determine the structures of organic compounds.^250,251^ While it is beyond the scope of this review to explore these methods in detail, many of these methods are applicable and/or adaptable to fluorine-containing molecules.

Data from NMR contain a wealth of information about the structures of the detected molecules. Moreover, the chemical shift range of ^19^F is large, resulting in good separation of resonances associated with various structural moieties (e.g., CF_3_ typically appears from ∼−60 to −90 ppm, while CF_2_ appears from −120 to −150 ppm). This good separation is expected to further improve at higher magnetic field strengths, at least for PFAS with molecular weights less than ∼1000 Da, such as PFOS (molecular weight of 500 Da).^1,19^ The high resolution obtained in a high magnetic field strength ^19^F NMR spectrum often enables the differentiation of PFAS from other forms of organic fluorine that do not meet the PFAS definitions, such as molecules with only one fluorine on a carbon, and inorganic fluorine, such as a fluoride ion, based on the chemical shifts of the resonances and the multiplet patterns. For example, fluoride ion appears as a sharp singlet at ∼−121 ppm in aqueous solution, making it relatively easy to identify, even in complex mixtures. Larger molecules, such as fluorinated proteins or fluoropolymers, result in increased line widths (e.g., peak broadening) due to enhanced relaxation associated with chemical shift anisotropy, making higher magnetic fields less beneficial because this relaxation increases as magnetic field strength increases.^252^ However, such peak broadening provides an important piece of evidence that either polymers are present in the sample or the compounds form larger complexes. Increasing the analysis temperature can increase molecular motion and offset these losses for larger molecules. Thus, ^19^F NMR may be a viable approach for the detection and quantification of water-soluble fluoropolymers that may make up a significant fraction of the organic fluorine in semiconductor wastewater^67^ and that cannot be easily determined by LC-HRMS approaches.

Simple total fluorine analysis is performed by integrating the fluorine spectrum and comparing the integrated area with that of the internal reference standard. Signals for the fluoride ion are separately integrated and subtracted from the total integral, resulting in total organic fluorine. It is also possible to identify specific PFAS in a mixture using libraries of chemical shifts to identify those that belong to the particular compound of interest.^248^ Similar techniques are used extensively in the metabolomics field with 1D ^1^H NMR, which has a much narrower chemical shift range (10 ppm) and consequentially increased resonance overlap compared to ^19^F NMR (150 ppm for most PFAS).^247^ Chemical shifts are a measure of the chemical environment of a nucleus, which is often impacted by the solvent, so libraries of chemical shifts should be obtained in the same solvent as the sample analysis to minimize the differences between the library and sample chemical shifts. At least one large NMR spectral library of PFAS is reported;^248^ however, care must be taken when using this library because the NMR spectra were collected in one of three solvents, chloroform, dimethyl sulfoxide, or methanol. Nevertheless, analysis of a firefighting aqueous film-forming foam sample using this library yielded the identities of several individual PFAS compounds.^248^ To the best of our knowledge, there is no comprehensive library of ^19^F NMR PFAS spectra collected entirely in water or methanol, which would be most relevant to the direct analysis of wastewater samples or to organic solvent extracts (e.g., SPE extracts) of wastewater, respectively. Also, *J*-coupling constants (spin–spin interactions that lead to peak multiplets) do not change with field strength; therefore, they will change when displayed on the chemical shift scale, which should be considered when comparing spectra collected at field strengths different from those in the library. Compounds previously identified by matching chemical shifts for the associated resonances against a chemical shift library are quantified using all of the matched resonances or using only a single resonance.^238,248^ If using only a single resonance for quantification, care must be taken that the resonance is correctly identified and that it does not overlap with resonances from other compounds. Using a single resonance alone for quantification may be desirable if the resonance is well resolved and unambiguously identified, and other resonances for the compound of interest are overlapped.

### 10.1 Increasing ^19^F NMR Sensitivity

The primary disadvantage of ^19^F NMR for PFAS analysis, especially for environmental matrices such as wastewater, is its low sensitivity. NMR is considered a low-sensitivity technique because the population differences between the ground and excited spin states that give rise to the NMR signal are small. The advent of high-field superconducting magnets and pulsed Fourier Transform NMR techniques provides substantial sensitivity boosts through increases in the ground/excited state population differences and signal averaging, respectively. The signal-to-noise achieved from a pulsed Fourier transform 1D NMR experiment depends on the number of scans performed, with the signal-to-noise ratio increasing with the square root of the number of scans.^253^ Thus, by increasing the number of scans and associated experimental time, the signal-to-noise ratio is increased; however, practical limits on available instrument time limit the gains possible from collecting more scans. The pulsed nature of NMR also makes stating explicit LOD dependent on the number of scans performed, which varies between studies. It is important to note that the LOD is expressed in the literature using various concentration units such as molar (M) or mass per unit volume (g/L). For NMR, the integrated area of a resonance is proportional to the number of nuclei contributing to the resonance, which is related to the molar concentration based on the molecular structure of the molecule. Therefore, in NMR, it is common for the LOD to be expressed in molar concentration units. Moreover, if the exact identity of a single compound is unknown, but a moiety of the compound (such as a CF_3_) is identified, then a molar concentration can still be determined, whereas a mass per volume (e.g., ng/L or μg/L) concentration cannot be unambiguously reported. The advent of cryogenic probes has further enhanced the signal-to-noise ratio by a factor of 2 to 4.^254^ Early cryoprobes were typically limited to ^1^H detection with the highest signal-to-noise, but more recent probes are able to tune to ^19^F, enabling large sensitivity gains for ^19^F NMR. Using an 800 MHz NMR spectrometer equipped with a ^19^F capable cryoprobe, a limit of quantification of 19 μM total fluorine (e.g., 520 μg/L PFOA) was achieved with approximately 30 min of acquisition, without additional steps to improve the signal-to-noise. While cryoprobes are considerably more sensitive than room temperature probes, they are also more sensitive to salt concentrations in the sample. High inorganic salt concentrations (e.g., >150 mM NaCl) can adversely impact the sensitivity of a cryoprobe and should be avoided when possible.^254^ In general, high salt concentrations, such as NaCl, that increase the sample conductivity will increase sample resistance, which results in a loss of signal-to-noise. The loss of signal-to-noise ratio is proportional to the conductivity of the sample. Different solutes, such as Cl^–^ vs PO_4_^2–^, also have different effects on conductivity, so different salt concentrations can result in different impacts on signal-to-noise.^254^ The effects of high salt are mitigated by changing the NMR tube diameter and geometry if removing the salt is not possible.^255^ Changes in the tube diameter and geometry reduce the total sample volume in the detection coil, which reduces the signal-to-noise ratio; therefore, these remedies should only be used when salt concentrations are high enough that the improvement gained from the change in geometry is larger than the loss from the smaller sample volume.

Additional optimizations of experimental parameters can further improve the signal-to-noise. For ^19^F detection, the relaxation delay between scans often needs to be long so that the nuclear spins return to equilibrium prior to the next radio frequency pulse, ensuring quantitative analysis. If the relaxation delay is too short and the nuclear spins do not return to equilibrium, the NMR signal will be saturated, and the resulting spectrum will no longer be quantitative. A recycle delay of 15 s is required for some quantitative ^19^F experiments at 800 MHz.^236,241^ A recent study showed that using a steady state pulse sequence combined with Complete Reduction to Amplitude Frequency Table (CRAFT)^256^ processing could significantly increase sensitivity; however, this approach was not quantitative.^257^ Previous studies show that including a paramagnetic relaxation agent, such as chromium­(III) acetylacetonate (Cr­(AcAc)_3_), enables much shorter relaxation delays (≤1 s), resulting in more scans per unit time and subsequently higher signal-to-noise.^237,239^ The concentration of a paramagnetic relaxation agent must be optimized to avoid resonances becoming overly broad. A concentration of Cr­(AcAc)_3_ of 4 mg/mL allowed a relaxation delay of less than 1s while still retaining good spectral resolution.^239^

Further improvements in the signal-to-noise ratio are achieved by concentrating the PFAS in the sample. A method based on SPE with an anion exchange sorbent was used to prepare PFAS-containing samples for NMR.^239,248,258^ Using a combination of relaxation agents and sample preconcentration, a LOD of 0.14 nM (16 ng/L) for TFA was obtained with approximately 2 h of acquisition on a typical room temperature-probe equipped 500 MHz spectrometer.^239^ Further enhancements to signal-to-noise ratios were proposed using additional statistical analysis of an array of NMR experiments collected on the same sample. Using this method with an approximately 6 h data acquisition, a relaxation agent, and a room temperature probe resulted in an LOD of 7.8 nM of an unknown PFAS, where quantification of the unknown compound was based on integration of the alkyl CF_3_ resonance alone.^243^ These enhancements to signal-to-noise have enabled the use of NMR to directly quantify the leaching of PFAS from several types of plastic tubing, some of which are commonly used in semiconductor manufacturing.^259^

### 10.2 Implications for Semiconductor Wastewater

Semiconductor manufacturing involves the use of inorganic (e.g., HF) and organic fluorine compounds, making the ability of NMR to differentiate between classes (e.g., aromatic, aliphatic, polymeric) of PFAS potentially invaluable. Inorganic fluorine may interfere with other analysis techniques such as those that rely on CIC but is easily identified and distinguished from organic forms of fluorine. Moreover, the ability of NMR to tolerate a wide range of sample conditions and solvents enables it to be used with both aqueous and organic solvents. NMR is also applicable to water-soluble fluoropolymers (MW greater than 1200 Da), which are found in antireflective coatings used in semiconductor manufacturing processes^1^ and are challenging for current MS-based methodologies. As noted above, increased sample temperatures may be employed for fluoropolymer analysis to reduce relaxation and improve signal quality.^245^ As noted previously, high salt/solute concentrations may reduce the signal-to-noise ratio due to increased sample resistance. For these samples, the experimental design will require optimization for the application. While NMR is often not severely impacted by the sample composition, the presence of high concentrations of paramagnetic ions will significantly enhance relaxation resulting in severe line broadening.^260^ Therefore, wastewaters with high (>1 mg/mL) concentrations of paramagnetic ions, such as Cu­(II) or Cr­(III), may be difficult or impossible to analyze with NMR if those ions are not first removed from the sample.

## 11 Sensors for Online Monitoring of PFAS in Wastewater

Methods, as described earlier, for analyzing target PFAS generally involve sample preparation, followed by LC–MS or GC–MS, which requires time, money, and trained laboratory staff to prepare and process samples. Alternatively, screening strategies using sensors could be deployed to prioritize samples that might then be analyzed for compliance purposes using conventional LC- or GC-based methods. Sensors could be used for online target PFAS monitoring as an early warning tool at wastewater treatment plants for rapid intervention. Alternatively, sensors could help to track down sources of PFAS along the unit of semiconductor manufacturing processes. Properties of PFAS, including the fact that they are neither UV–vis- or fluorescence-spectroscopically active nor electrochemically active, are problematic for direct measurement using traditional sensors that rely on spectroscopic or electrochemical detection. Therefore, indirect electrochemical methods have been developed that give better sensitivity over UV–vis or fluorescence indirect methods.^261,262^ The most promising electrochemical indirect method, in terms of sensitivity (<10 ppt) and selectivity, is based on the use of an electrode coated with a molecular-imprinted polymer (MIP). The MIP creates molecular-specific cavities that are similar in size, shape, and functional group of one specific target PFAS and consequently highly selective to this target PFAS.^263^ An other promising road is the development of direct quantum cascade laser mid-infrared sensor, which is able to measure target PFAS such as PFOA, but question remains in term of sensitivity and selectivity.^264^ Despite several submitted patents, the road to a commercial device remains at an early stage.^261,262^ The U.S. EPA’s PFAS action plan^265^ (EPA’s Small Business Innovation Research Program) is an assistance program for the development and support of promising, commercially available prototypes; for example, the technology 2Witech solution^266^ used MIP-based technology. Other promising prototypes based on indirect electrochemical methods involve electrically read lateral flow assay (e-LFA),^267^ metal–organic frameworks,^268^ and bubble-nucleation-based methods^269^ that exploit the surfactant properties of PFAS for measurement. In addition to sensitivity and selectivity, the stability of the sensor over time and the reliability of the technology needs to be improved before a commercial product is available on the market.

## 12 Conclusion

This review provides a comprehensive guide for wastewater sampling, nonvolatile and volatile PFAS analysis, total fluorine, and online monitoring with an emphasis on semiconductor wastewater. Feasibility to apply those methods to water exhaust of fabrication unit processes is unknown due to extreme pH condition and very high ionic strength, and this represents a research need. This review emphasizes the importance of considering the PFAS structure and properties to aid in understanding chemical behavior. Consequentially, knowing PFAS behavior aids in determining the most suitable analytical approaches for detection based on advantages and limitations of an instrument. This review also stresses the need for advanced techniques, including high-resolution mass spectrometry for suspect and nontarget analysis as well as nonspecific methods for total organic and inorganic fluorine.

While literature exists for nonvolatile PFAS occurrence in semiconductor wastewater, no comprehensive studies have been done to identify volatile PFAS, calculate total fluorine, or conduct mass balance. Additionally, future work is needed on conducting storage-stability tests for PFAS identified in semiconductor wastewater, expanding PFAS targets, Method 1633 (including ultrashort chain PFAS), and modifying total fluorine methods to account for high fluoride backgrounds in semiconductor wastewater. Furthermore, there is a need for exploration of alternative ionization techniques for poorly ionized PFAS for better detection and applying ^19^F NMR for quantifying total fluorine in semiconductor wastewater, including high molecular weight water-soluble fluoropolymers. By highlighting these research needs alongside current methods and workflows, this perspective aims to assist industry professionals and researchers in overcoming existing challenges. Advancing detection and environmental monitoring of PFAS emissions in semiconductor wastewater aids in the development of proper separation, and abatement processes can be developed to reduce PFAS concentrations and mitigate potential release.

## Supplementary Material



## Notes

Acronyms and glossary of term, structure of target and suspect PFAS, details/comparisons on methods, and nontarget overview (PDF)
