# Supplementary material for: Practical Guidance on Selecting Analytical Methods for PFAS in Semiconductor Manufacturing Wastewater
Source: ACS Meas Sci Au. 2025 Jun 20;5(4):399–423. doi: 10.1021/acsmeasuresciau.5c00021 (PMC12371593; doi:10.1021/acsmeasuresciau.5c00021)
Supplement: Supplementary file 1 [file tg5c00021_si_001.pdf]

Supporting Information

Practical Guidance on Selecting Analytical Methods for PFAS in Semiconductor Manufacturing Wastewater

Boris Droz[1], Christopher G. Heron[2], Mitchell L. Kim-Fu[3], Patrick N. Reardon[4], Mireia Roig-Paul[2], Jennifer A. Field[2,*]

[1]Department of Biological and Ecological Engineering, Oregon State University, Corvallis, OR 97331, USA

[2]Department of Environmental and Molecular Toxicology, Oregon State University, Corvallis, OR 97331, USA

[3]Department of Chemistry, Oregon State University, Corvallis, OR 97331, USA

[4]NMR Facility, Oregon State University, Corvallis, OR 97331, USA

[*]Corresponding Author

Email Address: jennifer.field@oregonstate.edu; Phone: (541) 737-2265

39 Pages

14 Tables

Text S1. Acronym.

ADONA: 4,8-dioxa-3H-perfluorononanoic acid

AOF: adsorbable organofluorine

ASTM: American Society for Testing and Materials

APCI: atmospheric pressure chemical ionization

APPI: atmospheric pressure photoionization

ASTM: American Society for Testing and Materials

CI: chemical ionization

CIC: combustion ion chromatography

DDA: data dependent analysis

DIA: data independent analysis

diPAP: dialkyl fluorotelomer phosphate diester

DOC: dissolved organic carbon

ECD: electron capture detector

ECF: electrofluorination

EI: electron impact ionization

EOF: Extractable organofluorine

EPA: Environmental Protection Agency

EtFOSA: *N*-ethyl perfluorooctane sulfonamide

EtFOSAA: *N*-ethyl perfluooooctane sulfonamido acetic acid

EtFOSE: *N*-ethyl perfluoroalkyl sulfonamido ethanol

FTOH: fluorotelomer alcohols

FASAs: perfluoroalkyl sulfonamides

FOSA: perfluorooctane sulfonamides

FOSE: perfluorooctane sulfonamido ethanol

FTACs: fluorotelomer acrylates

FTI: fluorotelomer iodides

FTIR: Fourier-transform infrared spectroscopy

FT-ICR-MS: Fourier-transform ion cyclotron resonance mass spectrometry

FTMACs: fluorotelomer methacrylates

FTO: fluorotelomer olefins

FTOH: fluorotelomer alcohols

FTS: fluorotelomer sulfonates

GC: gas chromatography

GCB: graphitized carbon black

GF-MAS: graphite furnace molecular adsorption spectrometry

HF: hydrogen fluoride

HFPO-DA: 2,3,3,3-tetrafluoro-2-(1,1,2,2,3,3,3-heptafluoropropoxy)propanoic acid

HILIC: hydrophobic interaction chromatography

HLB: hydrophilic-lipophilic balanced

HRMS: high-resolution mass spectrometry

HS: headspace

INAA: Instrumental neutron activation analysis

KMD: Kendrick mass defect

LC-MS/MS: liquid chromatography tandem mass spectrometry

LOD: limit of detection

LOQ: limit of quantification

MD: mass defect

MeFOSA: *N*-methyl perfluorosulfonamide

MeFOSAA: *N*-methyl perfluoooctane sulfonamido acetic acid

MeFOSE: *N*-methyl perfluoroalkyl sulfonamido ethanol

MRM: multiple reaction monitoring

NaCl: sodium chloride

$NaNO_3$: sodium nitrate

NMR: nuclear magnetic resonance

MIP: molecular-imprinted polymer

MPFAS: stable isotope PFAS added prior to extraction

M2PFAS: stable isotope PFAS added after sample preparation

MRM: multiple reaction monitoring

MS1: accurate precursor mass

MS2: molecular fragments

OECD: Organisation for Economic Cooperation and Development

PCI: positive chemical ionization

PDMS: polydimethylsiloxane

PFAS: Per- and polyfluoroalkyl substances

PFBA: perfluorobutanoic acid

PFBS: perfluorobutane sulfonate

PFCAs: perfluoroalkyl carboxylates

PFEAs: polyfluoroalkyl ether acids

PFAI: perfluoroalkyl iodides

PFPrA: pentafluoropropanoic acid

PFPrS: perfluoropropane sulfonate

PFSAs: perfluoroalkyl sulfonates

PFOA: perfluorooctanoic acid

PFOS: perfluorooctane sulfonic acid

PIGE: particle induced gamma ray emission spectroscopy

pKa: acid dissociation constant

QTOF: quadrupole time of flight

RP: reverse phase chromatography

SBSE: stir-bar sorptive extraction

SFC: supercritical fluid chromatography

SPE: solid phase extraction

SPME: solid phase microextraction

TARC: top antireflective coatings

TD: thermal desorption

TF: Total Fluorine

TFA: trifluoroacetic acid

TFMS: trifluoromethane sulfonate

TOF: time of flight (mass spectrometer)

TOP (assay): Total oxidizable precursor assay

TQ: triple quadrupole

U.S. EPA: United States Environmental Protection Agency

WAX: weak anion exchange

Text S2. Glossary.

Accurate mass – measure of the mass of a PFAS into the mass spectrometer.

Class of PFAS – PFAS having similar chemical structures.

Feature – a distinct m/z and retention time pair (mz@RT) that represent an individual compound.

Fragmentation – process of breaking a PFAS ion analyzed by the MS1 into piece of fragment containing part of original structure of the PFAS.

Ionogenic – molecule capable of ionizing.

Kendrick mass defect – The Kendrick mass (KM) value is calculated by multiplying the accurate mass (m/z) of a PFAS by the rounded R value (the nearest integer value of a repeating unit (R) mass, such as 50 for CF2 or 32 for CFH) divided by the exact R value. Then the Kendrick mass defect (KMD) is KM subtracted from the rounded KM .

$$\mathrm{KM} = \frac{\mathrm{m}}{\mathrm{z}} \times \frac{\mathrm{round(R)}}{\mathrm{R}}$$

$$\mathrm{KMD} = \mathrm{round(KM)} - \mathrm{KM}$$

Long-chain PFAS – PFAS containing more than eight fluorinated carbon atoms.

Mass accuracy – the difference between accurate mass and exact mass of a molecule. Typically, below 5 ppm in HRMS.

Mass defect – m/z – rounded(m/z). A mathematical subtraction where m/z is the accurate mass to charge of an analyte.

Nontarget PFAS – previously unidentified PFAS.

Passive sampler – Integrative sampler made of a diffusion cell with some sorbent material providing a time-integrate concentration of the PFAS in the environment.

Precursor ion – terms for PFAS involved in fragmentation reactions into the collision chamber (MS2) of the mass spectrometer.

Pyrolyze – thermal decomposition of a compound under controlled environments, typically under limiting amount of oxygen.

Resolution – ratio of mass of the PFAS of interest and the mass difference (≥20,00 for HRMS; unitless) is the ability of an instrument to distinguish two peaks of slightly different mass-to-charge (m/z) ratios.

Response factor – ratio between the signal, e.g., area count, measured by the instrument and the quantity of the PFAS which produced the signal. Response factors are specific for each PFAS and are determined by injecting a standard.

Scan speed – time require for the acquisition of one MS2 spectra.

Short-chain PFAS – PFAS containing between five to seven fluorinated carbon atoms.

Suspect PFAS –previously identified PFAS but with no commercial analytical standard.

Target PFAS – PFAS for which analytical standards are available commercially for purchase.

Treated semiconductor wastewater – wastewater treated onsite using physical, chemical, and/or biological treatment to meet national, local, and company-specific effluent discharge requirements, before discharge to local wastewater treatment facilities or surface water.

Ultrashort-chain PFAS – PFAS containing up to four fluorinated carbon atoms.

Wall-Coated Open Tubular Column – a 0.1–0.5 μm film of a liquid stationary phase coating the inner wall of a fused silica capillary coated with polyimide.

Workflow – sequential series of process and steps that can be repeat automatically or manually to analyze data. This can have different software interface like an open-source code, a web-based package application or a window panel software.

Table S1. Target **ionogenic/nonvolatile** PFAS included in EPA and ASTM methods that are potentially applicable for semiconductor wastewater: green = little modification and yellow = requires significant modification. Proton on ionizable functional group denoted by red H.

Class	Structure	(n)	Name (Acronym)	EPA Method				ASTM		DIN	ISO
				537.1	533	1633	3512/ 8327	D7979-20	D8421-25	38407-42	21675:2019
PFCA	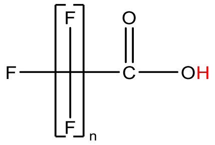	3	Perfluorobutanoic acid (PFBA)								
		4	Perfluoropentanoic acid (PFPeA)								
		5	Perfluorohexanoic acid (PFHxA)								
		6	Perfluoroheptanoic acid (PFHpA)								
		7	Perfluorooctanoic acid (PFOA)								
		8	Perfluorononanoic acid (PFNA)								
		9	Perfluorodecanoic acid (PFDA)								
		10	Perfluoroundecanoic acid (PFUnDA)								
		11	Perfluorododecanoic acid (PFDoA)								
		12	Perfluorotridecanoic Acid (PFTrDA)								
		13	Perfluorotetradecanoic acid (PFTeDA)								
PFSA	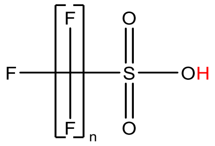	4	Perfluorobutanesulfonic acid (PFBS)								
		5	Perfluoropentanesulfonic acid (PFPeS)								
		6	Perfluorohexanesulfonic acid (PFHxS)								
		7	Perfluoroheptanesulfonic Acid (PFHpS)								
		8	Perfluorooctanesulfonic acid (PFOS)								

Class	Structure	(n)	Name (Acronym)	EPA Method				ASTM		DIN	ISO
				537.1	533	1633	3512/ 8327	D7979-20	D8421-25	38407-42	21675:2019
[illegible]	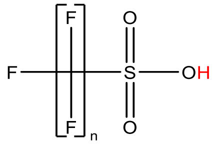	9	Perfluorononanesulfonic acid (PFNS)								
		10	Perfluorodecanesulfonic acid (PFDS)								
		12	Perfluorododecanesulfonic acid (PFDoS)								
PFecHS	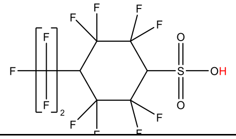	2	Decafluoro-4-(pentafluoroethyl) cyclohexanesulfonic acid								
n:2 FTS	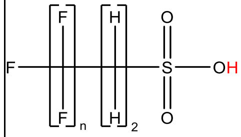	4	1*H*,1*H*,2*H*,2*H*-Perfluorohexane sulfonic acid (4:2FTS)								
		6	1*H*,1*H*,2*H*,2*H*-Perfluorooctane sulfonic acid (6:2 FTS)								
		8	1*H*,1*H*,2*H*,2*H*-Perfluorodecane sulfonic acid (8:2FTS)								
FOSA	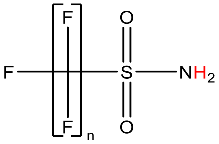	8	Perfluorooctane sulfonamide								
NMeFOSA	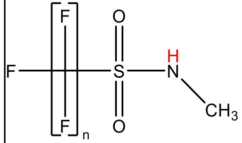	8	*N*-Methyl perfluorooctane sulfonamide								
NEtFOSA	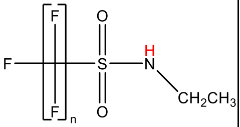	8	*N*-Ethyl perfluorooctane sulfonamide								

Class	Structure	(n)	Name (Acronym)	EPA Method				ASTM		DIN	ISO
				537.1	533	1633	3512/ 8327	D7979-20	D8421-25	38407-42	21675:2019
NMeFOSAA	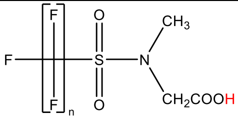	8	*N*-methyl perfluorooctane sulfonamidoacetic acid								
NEtFOSAA	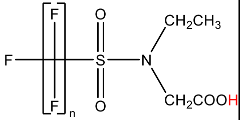	8	*N*-ethyl perfluorooctane sulfonamidoacetic acid								
NMeFOSE	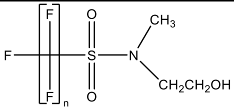	8	*N*-Methyl perfluorooctane sulfonamidoethanol								
NEtFOSE	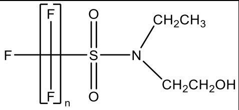	8	*N*-Methyl perfluorooctane sulfonamidoethanol								
HFPO-DA	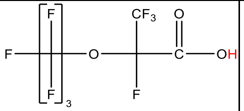	n/a	Hexafluoropropylene oxide dimer acid								
ADONA	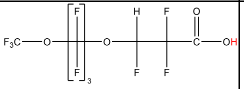	n/a	4,8-Dioxa-3*H*-perfluorononanoic acid								
PFMPA	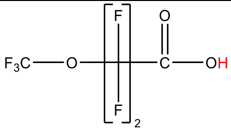	n/a	Perfluoro-3-methoxypropanoic acid								

Class	Structure	(n)	Name (Acronym)	EPA Method				ASTM		DIN	ISO
				537.1	533	1633	3512/ 8327	D7979-20	D8421-25	38407-42	21675:2019
PFMBA	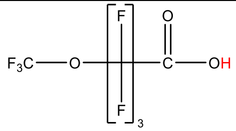	n/a	Perfluoro-4-methoxybutanoic acid		■	■			■		
NFDHA	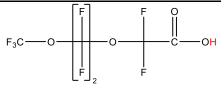	n/a	Nonafluoro-3,6-dioxaheptanoic acid		■	■			■		
9Cl-PF3ONS	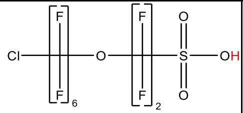	n/a	9-Chlorohexadecafluoro-3-oxanonane-1- sulfonic acid	■	■	■			■		■
11Cl-PF3OUdS	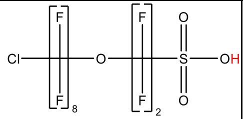	n/a	11-Chloroeicosafluoro-3-oxaundecane-1- sulfonic acid	■	■	■			■		
PFEESA	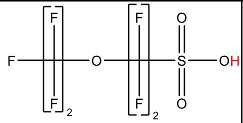	n/a	Perfluoro(2-ethoxyethane)sulfonic acid		■	■			■		
n:3 FTCA	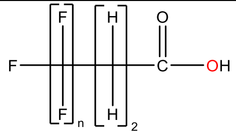	3	3-Perfluoropropyl propanoic acid (3:3FTCA)			■			■		
		5	*2H,2H,3H,3H*-Perfluorooctanoic acid (5:3FTCA)			■			■		
		7	3-perfluoroheptyl propanoic acid (FHpPA or 7:3FTCA)			■		■	■		

Class	Structure	(n)	Name (Acronym)	EPA Method				ASTM		DIN	ISO
				537.1	533	1633	3512/ 8327	D7979-20	D8421-25	38407-42	21675:2019
n:2 FTCA	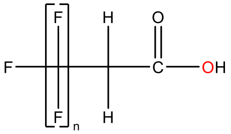	6	2-perfluorohexyl ethanoic acid (FHEA)					■			
		8	2-perfluorooctyl ethanoic acid (FOEA)					■			
		10	2-perfluorodecyl ethanoic acid (FDEA)					■			
n:2 FTUCA	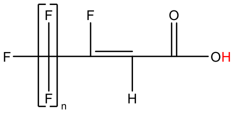	6	2*H*-perfluoro-2-octenoic acid (FHUEA)					■	■		
		8	2*H*-perfluoro-2-decenoic acid (FOUEA)					■	■		■

Table S2. Structures presented in Jacobs et al.[1] and their potential for analysis by US EPA Method 1633 and LC-MS/MS or HRMS: green = little modification and yellow = requires significant modification. Proton on ionizable functional group denoted by red H.

Structure	Class*	n=	Potential to add to Method 1633
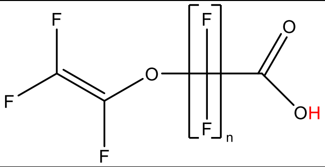	1**	n=1-3	
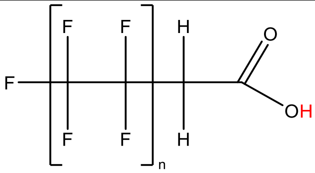	S-1	n=3,4	
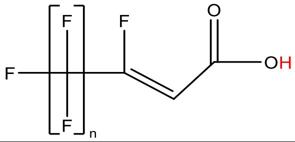	2	n=3,5-7	
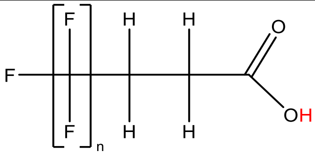	3	n=5-7	
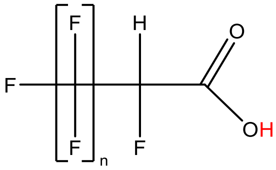	4**	n=2-5	
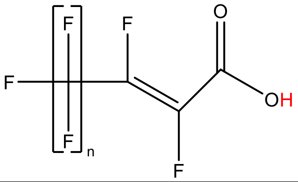	5	n=3-8	
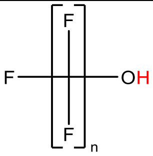	6	n=2-4	Ultra-short chain
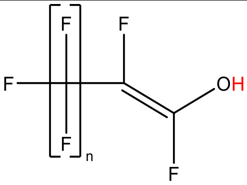	7**	n=2-4	

Structure	Class*	n=	Potential to add to Method 1633
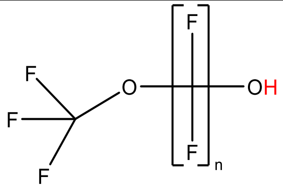	8	n=4-6	
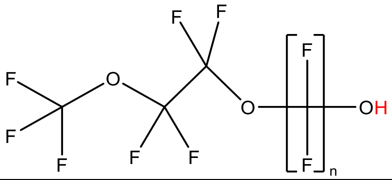	9	n=3-6	
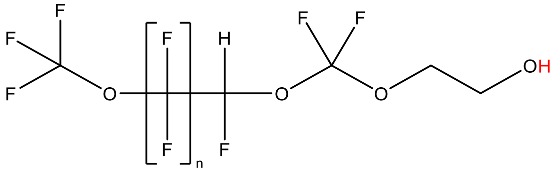	10	n=0,1	Neutral molecule, adduct
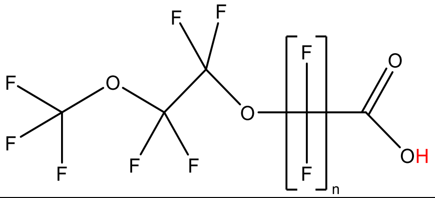	11	n=1-3	
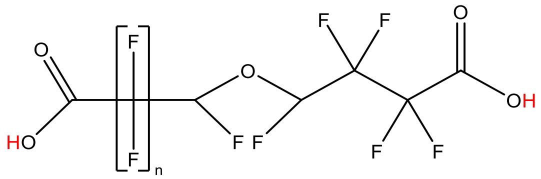	12**	n=2-5	
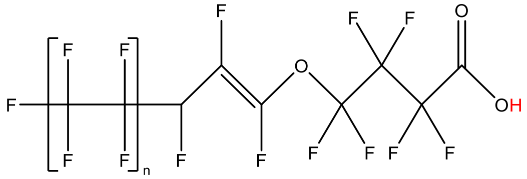	13**	n=1-3	
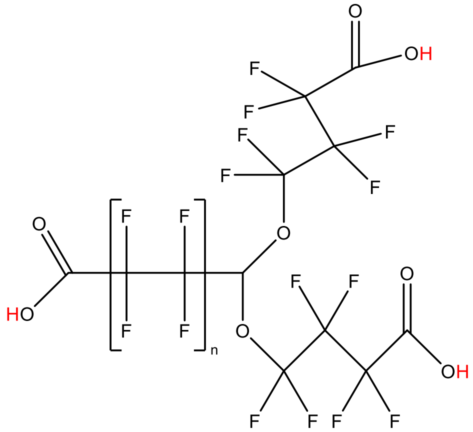	14**	n=1-3	

Structure	Class*	n=	Potential to add to Method 1633
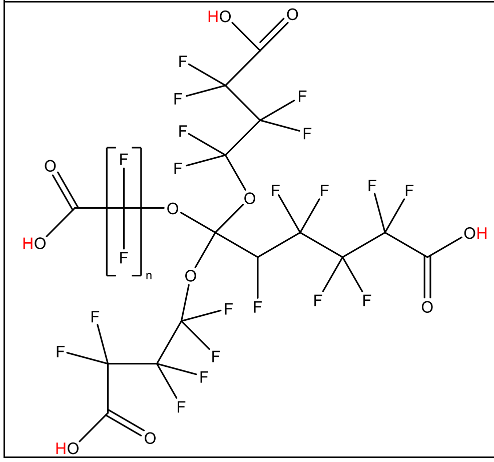	15**	n=3-7	

*Class refers to the original paper class used from Jacobs et al.[1] ** found in greatest abundance.

Table S3. Suspect PFAS structures found in semiconductor wastewater[2,3] and their potential for analysis by EPA Method 1633 and LC-MS/MS or LC-HRMS: green = little modification and yellow = requires significant modification. Proton on ionizable functional group denoted by red H.

Structure	Class*	n=	Potential to add to Method 1633
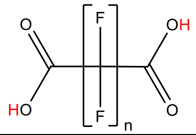	2	n=2-8	Some homologs are ultra-short chain
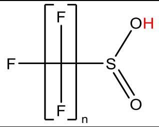	4	n=4	
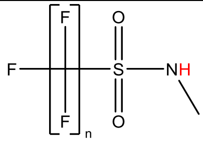	6	n=4	
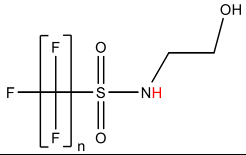	7	n=2-4	
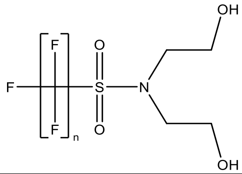	8	n=4	Neutral molecule, adduct
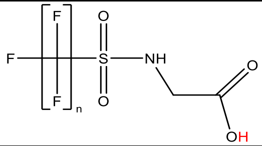	9	n=4	
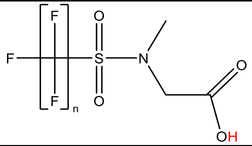	10	n=4	
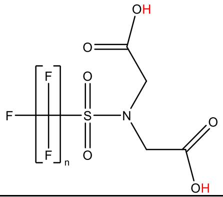	11	n=4	
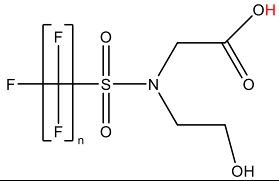	12	n=4	

*Class refers to the original paper class used in the original paper. [2,3]

Table S4. Structures presented in Supplemental Litho Examples and their potential for analysis by US EPA Method 1633 with LC-MS/MS or LC-HRMS: green = little modification; yellow = requires modification; orange = neutral PFAS would require significant modification/not suitable. Rf not defined. Proton on ionizable functional group denoted by red H.

Structure	Potential to add to Method 1633	Ref.
CF_3—SO_3H	Ultra-short-chain PFAS	4
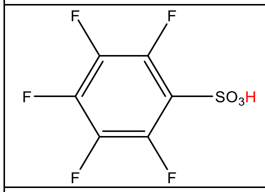		5
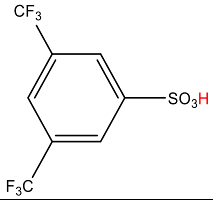		6
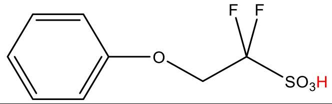		7
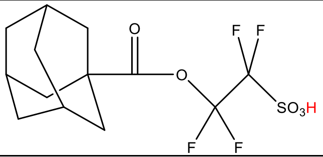		8
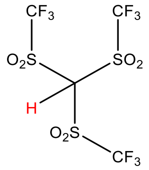		9
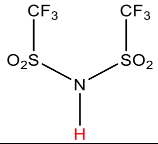		9
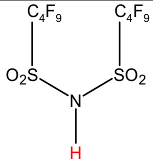		9
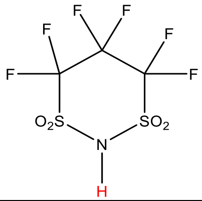		10

Structure	Potential to add to Method 1633	Ref.
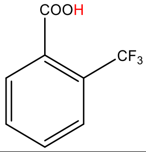		11
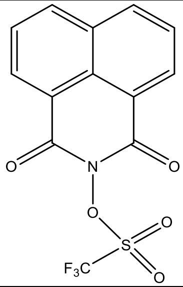	Neutral PFAS	12
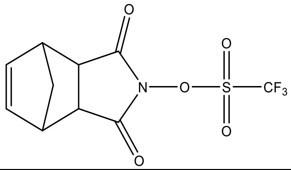	Neutral PFAS	13
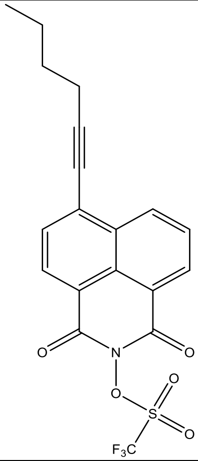	Neutral PFAS	14
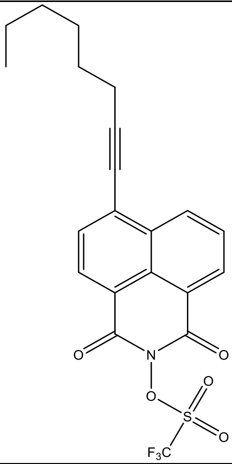	Neutral PFAS	15

Structure	Potential to add to Method 1633	Ref.
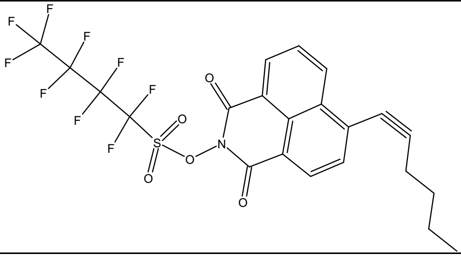	Neutral PFAS	15
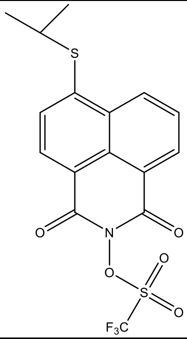	Neutral PFAS	14
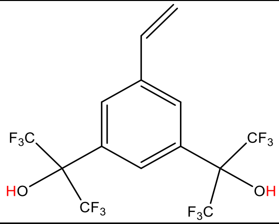	Depends on pKa of alcohol	16
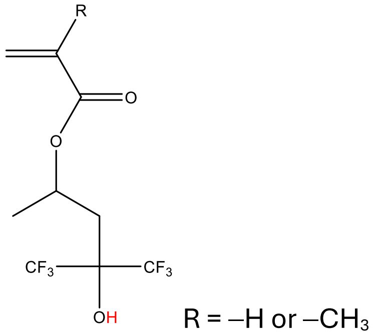	Depends on pKa of alcohol	16

Table S5. Structures (non-target PFAS) presented in Ober et al., [4] and their potential for analysis by US EPA Method 1633 and LC-MS/MS or LC-HRMS: green = little modification; yellow = requires modification; orange = requires significant modification/not suitable. Proton on ionizable functional group denoted by red H.

Structure	Chemical or Fig No. in Original Ref[4]	Potential to add to Method 1633
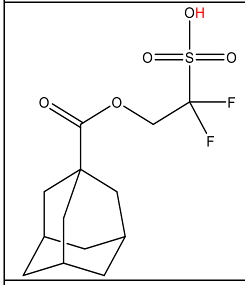	Fig 1	
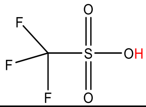	Fig 2,5,6	Ultra-short chain
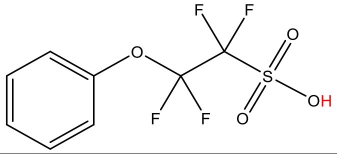	Fig 1	
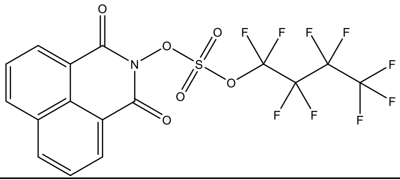	Fig 9	Neutral PFAS
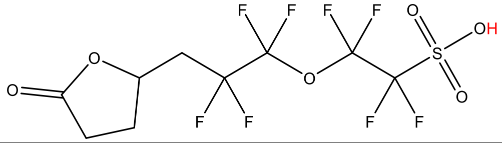	Fig 9	
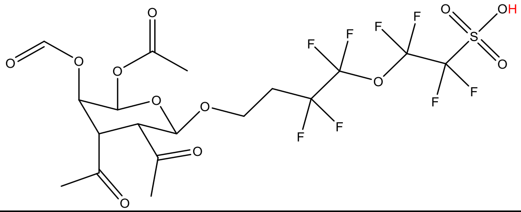	Fig 9	
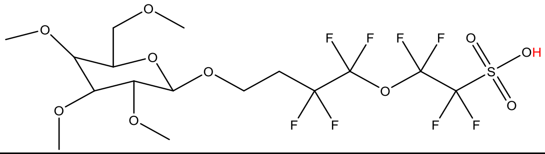	Fig. 9	
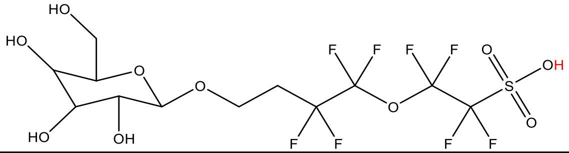	Fig. 9	

Structure	Chemical or Fig No. in Original Ref[4]	Potential to add to Method 1633
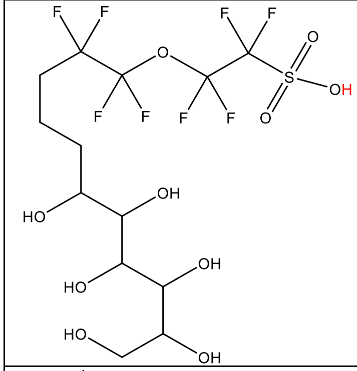	Fig. 9	
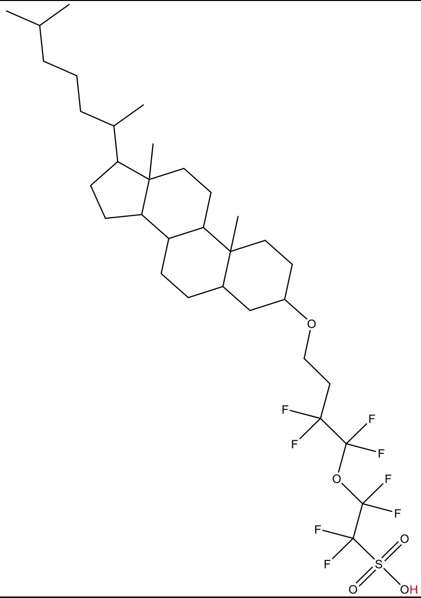	Fig. 9	
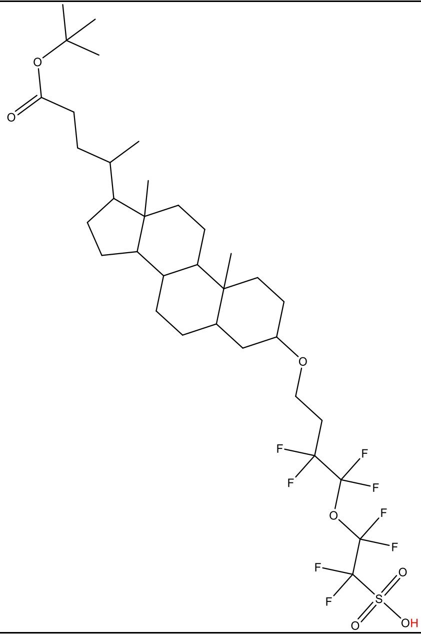	Fig. 9	

Table S6. Structures (suspect PFAS) provided by additional papers.[17] Proton on ionizable functional group denoted by red H.

Structure	Name (Acronym)	Chemical or Fig No. in Original Reference	Potential to add to Method 1633
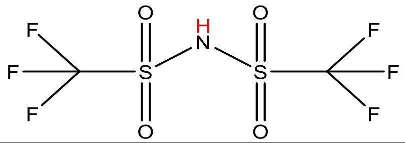	bistriflimide	18,19	

Table S7. Comparison guide for US EPA methods for PFAS in semiconductor wastewater.

<table>
<tr><th></th><th>EPA Method 537.1</th><th>EPA Method 533</th><th>EPA Method 1633</th><th>EPA Method 3512/8327</th><th>ASTM D7979-20</th></tr>
<tr><td>Suitable for Wastewater</td><td>No
(drinking water)</td><td>No
(drinking water)</td><td>yes</td><td>yes</td><td>yes</td></tr>
<tr><td>Numbers of. Target PFAS/MPFAS/M2PFAS</td><td>18/3/3</td><td>25/163/3</td><td>40/24/7</td><td>24/19/NA</td><td>21/9/NA</td></tr>
<tr><td>MPFAS[1] Name</td><td>Surrogate Analyte</td><td>Isotope Dilution Analogues</td><td>Extracted Internal Standard (EIS)</td><td>Surrogates</td><td>Surrogate Standard</td></tr>
<tr><td>M2PFAS[2] Name</td><td>Internal Standard</td><td>Isotope Performance Standard</td><td>Non-Extracted Internal Standard (NIS)</td><td colspan="2">NA</td></tr>
<tr><td>Sample prep</td><td>Reverse phase SPE</td><td>Mixed-Mode Polymeric</td><td>WAX SPE</td><td>Solvent Dilution</td><td>Direct Injection</td></tr>
<tr><td>Advantages</td><td>Commercially available</td><td>Commercially available
Isotope dilution</td><td>Commercially Available
Isotope dilution
Validated for wastewater</td><td colspan="2">Simple
Cost effective
Validated for wastewater</td></tr>
<tr><td rowspan="2">Limitations</td><td colspan="2">Not intended or validated for wastewater</td><td rowspan="2">More expensive</td><td colspan="2" rowspan="2">Limited commercial availability
External calibration
High detection limits</td></tr>
<tr><td>Not Isotope Dilution</td><td></td></tr>
<tr><td>Cost Range[3]</td><td>$</td><td>$$</td><td>$$$</td><td>$</td><td>$</td></tr>
</table>

[1]MPFAS – first stable isotope-labelled standard
[2]M2PFAS – second stable-isotope labelled standard
[3]Relative indicator of the price
NA not applicable

Table S8. Methods for ultra-short chain (<C4) perfluoroalkyl carboxylates (PFCAs) and perfluoroalkyl sulfonates (PFSAs) with example limits of quantification (LOQ) for trifluoroacetic acid (TFA) and trifluoromethane sulfonate (TFMS).

**Preconcentration**	**Analytical Column**	**Instrument**	**Sample volume (mL)**	**LOQ (ng/L)**	**Ref.**
Evaporation and derivatization	DB-17	GC-MS	500–1000	1 TFA	[20-24]
Weak anionic exchange SPE	HILIC and reverse phase (RP) HSS T3	LC-MS/MS	100–200	0.5 TFMS	[25]
	RP Kinetex C18		50	15–26 TFA**	[26]
	RP HSS T3		200–250	None provided	[3,17]
	RP RSpak JJ-50		100–200	0.5 TFA	[27]
	NP Diol	SFC-MS/MS	50–200	25–200 TFA	[28,29]
	Raptor Polar X*	LC-HRMS	50–500	Not provided	[30]
Mixed mode SPE	HILIC		100–200	Not provided	[31,32]
	WAX-1*		500	64 TFA***	[33]
			5	5–10 TFA	[1]
HLB SPE	HILIC		100–200	Not provided	[31]
Direct injection	NP Diol	SFC-MS/MS	0.25	34 TFA	[34]

* Raptor Polar X and WAX-1 combine hydrophobic and ion-exchange retention; **LOQ was measured for different water matrix; *** method quantification limits (MQL) is provided instead of the LOQ; acronyms used in the table are defined in Text S1.

Table S9. Volatile PFAS for which analytical standards are commercially available.

Class	Structure	Homolog (n)	Name (Acronym)
n:2 FTOH Fluorotelomer alcohol	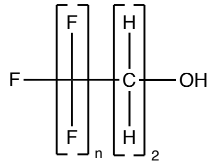	1	1:2 FTOH
		2	2:2 FTOH
		4	4:2 FTOH
		6	6:2 FTOH
		8	8:2 FTOH
		10	10:2 FTOH
		12	12:2 FTOH
n:1 FTOH Fluorotelomer alcohol	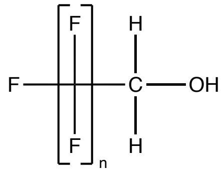	2	2:1 FTOH
		3	3:1 FTOH
		5	5:1 FTOH
		6	6:1 FTOH
		7	7:1 FTOH
		8	8:1 FTOH
		9	9:1 FTOH
		10	10:1 FTOH
		13	13:1 FTOH
n:3 FTOH Fluorotelomer alcohol	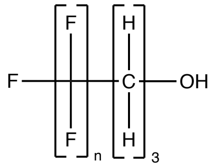	1	1:3 FTOH
		3	3:3 FTOH
		6	6:3 FTOH
		8	8:3 FTOH
Branched fluorotelomer alcohol	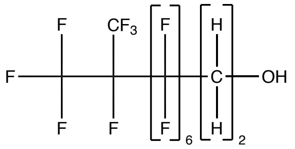	-	9Me 8:2 FTOH
Allylic fluorotelomer alcohol	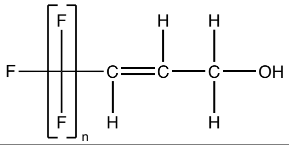	6	Allylic 6:3 FTOH
Fluorotelomer dialcohol	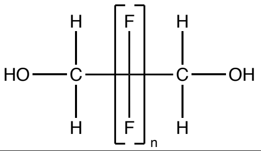	8	1:8:1 diFTOH

Class	Structure	Homolog (n)	Name (Acronym)
FTAC Fluorotelomer acrylate	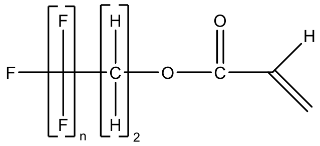	6	6:2 FTAC
		8	7:2 FTAC
		10	10:2 FTAC
FTMAC Fluorotelomer methacrylate	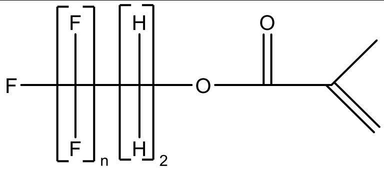	6	6:2 FTMAC
		8	8:2 FTMAC
n:2 FTO Fluorotelomer olefin	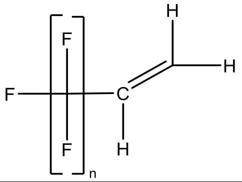	6	6:2 FTO
		8	8:2 FTO
		10	10:2 FTO
		12	12:2 FTO
FTI Fluorotelomer iodide	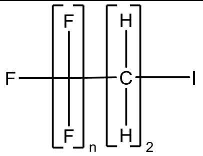	4	4:2 FTI
		6	6:2 FTI
		8	8:2 FTI
		10	10:2 FTI
PFAI Perfluoroalkyl iodide	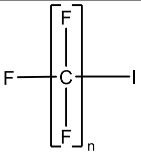	6	PFHxI
		8	PFOI
		10	PFDI
FTAT Fluorotelomer acetate	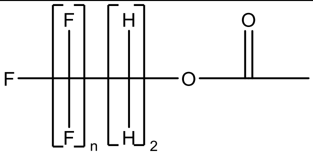	8	8:2 FTAT
		10	10:2 FTAT
sFTOH secondary fluorotelomer alcohol	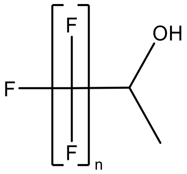	3	3:2 sFTOH
		5	5:2 sFTOH
		7	7:2 sFTOH
Perfluoro ketone	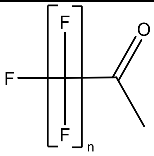	5	-
		6	-
		8	-
n:2 FTAL Fluorotelomer aldehyde	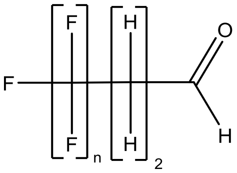	8	8:2 FTAL

Class	Structure	Homolog (n)	Name (Acronym)
FASA	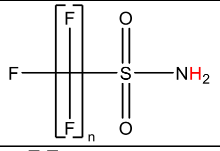	8	FOSA
N-methyl perfluorooctane sulfonamide	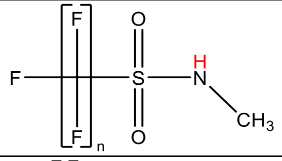	8	MeFOSA
N-ethyl perfluorooctane sulfonamide	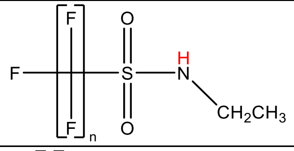	8	EtFOSA
N-methyl perfluoroalkyl sulfonamido ethanol	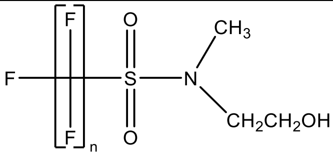	8	MeFOSE
EtFOSE *N*-methyl perfluoroalkyl sulfonamido ethanol	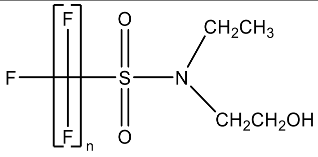	8	EtFOSE

Table S10. Methods sample preparation for volatile PFAS in wastewater.

Phase	Sample Preparation Method	Advantages	Limitations	Ref.
Liquid	Solid Phase Extraction (SPE)	Extract undergoes LC-MS or GC-MS analysis, concentrates large volume of water	Eluting solvent amount should be kept to a minimum to maximize LODs, solvent evaporation not recommended due to potential loss of volatiles	[35-37]
	Stir Bar Sorption Extraction (SBSE)	Minimal sample preparation, low method detection limits (ng/L)	Requires thermal desorption unit, only been demonstrated for few volatile PFAS targets in water	[38]
Gas	Purge and Trap	Either done online with GC-MS (no solvent needed) or to an SPE cartridge, minimal sample preparation	Requires specialized instrument for on-line purge and trap with GC-MS, eluting solvent should be kept to a minimum if not using online approach	[37,39,40]
	Head Space-Solid Phase Microextraction (HS-SPME)	No solvent needed to extract volatile PFAS from fiber (direct desorption to GC-MS), minimal sample preparation	Matrix backgrounds may result in competition for adsorption to fiber, carryover possible if not fully desorbed	[41,42]

Table S11. Detectors for volatile PFAS in wastewater.

Separation Method	Detector/Ionization Source	Advantages	Limitations	Ref.
Gas Chromatography	Electron capture detector	Halogen (e.g., F) detection	Target PFAS only, detects all halogenated compounds, lacks selectivity (no structural information)	42-44
	Chemical ionization mass spectrometry	Enhanced molecular ion (soft ionization), more selective: molecular weight confirmation	Less structural information (decrease in fragments), difficult for library matching	45-47
	Electron ionization mass spectrometry	Enhanced fragmentation (hard ionization), library matching for suspect PFAS	Limited molecular ions, difficult to differentiate PFAS with similar fragmentation	45
Liquid Chromatography	Electrospray ionization mass spectrometry	Detects some volatile PFAS classes as adducts	Adducts only stable at lower interface temperatures, most volatile PFAS poorly ionized, mobile phase impacts ionization	48-50
	Atmospheric pressure or chemical Ionization	More classes of volatile PFAS detected compared to LC-ESI-MS	Mobile phase impacts ion intensity, sensitive to tube lens offset voltage, large background	36,45,51

Table S12. Mode of mass fragmentation (MS2) acquisition and their advantages and limitations.

Mode of Acquisition	Advantages	Limitations
Data-Dependent Acquisition (DDA)	Good quality MS2 spectra but only on the most abundant features	Low abundant features may not be selected for mass fragmentation, may go undetected/unidentified due to the lack of MS2 data
Data-Independent Acquisition (DIA)	Generate MS2 for all precursor ions	Complex data processing (i.e., deconvolution) requires some advance data processing
Multiple Reaction Monitoring (MRM)	MS2 spectra with the highest quality and highest sensitivity (optimal for quantification)	Should know a-priori the retention time window of interest

Table S13. Overview of the nontarget workflow steps and limitations.

Precursor ion	Application	Limitations
Mass defect (MD)	Prioritizes high F content (F/C > 0.8, H/F < 0.8, mass percent of fluorine > 55%) PFAS compared to other organic compounds. Range: −0.49 to 0.50 Da for known PFAS	MD range overlaps with other organic compound limited usability for PFAS with low F-content. Halogens (e.g., Cl and Br) can also lead to negative MDs.
Mass defect/Carbon	Higher accuracy prioritization strategy than MD. Feature close to −0.003 MD/C and m/C close to 50, indicates a potential fluorinated compound	Need accurate ^{13}C isotope abundance estimate for each feature to calculate C content. Very low or very high intensities signals lead to high C number uncertainty. Like the MD, limited to high F-content PFAS
Kendric mass defect (KMD)	Prioritization and assignment of a homologue series where value is diagnostic of the series. KMD values are between −0.5 to 0.5	Occurrence of several members of a homologous series needed. Fewer than 3 homologs in a series may be false positive
Suspect screening	Assignment of a PFAS structure based on mass and retention time (optional)	High rate of false positive assignments if used alone. Need suspect list which are limited to already known PFAS
Chemical formula assignment	Reduces the number of potential chemical candidate using a given elemental composition	Omit compounds which do not match with the range of searched element. Number of potentials formula found is generally high and could not be conclusive

MS2 Fragments	Application	Limitations
Diagnostic fragment	Gives some structural information for compound identification on the fluorinated chain length and/or functional group present.	Limited detection for low abundance MS2 ions. No exhaustive lists of PFAS diagnostic fragments currently available.
Fragment difference & neutral losses	Usually applied together with diagnostic fragment. Gives information on the PFAS chain length. Advantage: PFAS transformation products that conserve molecular moieties.	MS2 not always available for low abundance ions. Very sensitive to mass accuracy to minimize false-positive detection.
Fragmentation flagging	Selective for compounds with specific fragments.	In-source fragmentation requires knowledge on source parameters values, may be limited to PFAS that easily form in-source fragments. Under DIA, data complexity might limit this approach to most abundant ions.
MS2 library search	Easy to apply, no previous knowledge required.	Matching metrics used by proprietary software may result in false positive/negative when low matching score (<90%). Match score depends on data quality and available spectra present on the library.
In-silico MS2 spectra	Compare unknown MS2 with potential structure, then the on-silico MS2 spectra can be stored and used as a MS2 library.	High computing demand. Need calibrated algorithm to compute new in-silico MS2 spectra.

Several vendor or open-source software exist to perform nontarget analysis where each software might generally contain a portion of the tools presented in this table. Comparison of the different software capabilities could be found elsewhere.[52-57]

Table S14. Comparison of nonspecific methods for PFAS detection.

Method	Sample Preparation	Detection Method	Limitations of Method	Method Detection Limit (ng F/L)	Ref.
TOP	1. Split aqueous samples into two, oxidize one of half, second half unoxidized – both fractions concentrated by SPE<br>2. Concentrate sample by SPE, split extracts, one fraction is oxidized, second fraction not oxidized	LC-MS/MS	Limited structural information, unknown oxidation pathway, requires two analyses by LC-MS/MS, unknown oxidation potential of PFAS used by semiconductor industry	1–2*<br>100–500*<br>0.009–0.217 ≥C4, 25 <C4*¥<br>10–100**¥<br>0.005–2.62*¥<br>0.002–0.28**¥<br>90–1320**¥<br>31–110**¥<br>2–37*¥<br>0.1–3.7*¥	58<br>59<br>29<br>60<br>61<br>62<br>63<br>64<br>65<br>66
EOF	WAX/ EnviCarb SPE column, elute, combust eluant	CIC	Ultrashort chains poorly recovered, polyfluoroethers not tested	300–500*<br>6–55*<br>25–43.5*<br>7–4300**<br>300*<br>10,000*	67<br>68<br>29<br>69<br>70<br>71
AOF	Load sample onto activated carbon SPE column, wash fluoride, combust activated carbon column	CIC	Washing steps remove ultrashort chain PFAS, however washing is necessary for fluoride removal; polyfluoroethers not tested.	1500–2900*<br>50*<br>770**<br>200–500*<br>300*<br>2000**<br>20000**<br>10,000*<br>50–250*	72<br>73<br>74<br>67<br>75<br>76<br>77<br>71<br>78
	Carbon fiber sorbent	PIGE	Does not distinguish between fluorine and fluoride	50*	79
TF	Place sample directly onto sample boat for combustion	CIC	No fluoride removal, may overestimate concentration	20000**	77

Method Detection Limit is referred as *limit of detection (LOD) or **limit of quantification (LOQ) LOD ¥refer as ng/L of perfluoroalkyl carboxylates (PFCAs) instead of F. Method are TOP (assay): Total oxidizable precursor assay, EOF: Extractable organofluorine AOF: adsorbable organofluorine and TF: Total fluorine. Detection method are LC-MS/MS: liquid chromatography tandem mass spectrometry, CIC: combustion ion chromatography and PIGE: particle induced gamma ray emission spectroscopy.

LITERATURE CITED

(1) Jacob, P.; Helbling, D. E. Rapid and simultaneous quantification of short- and ultrashort-chain perfluoroalkyl substances in water and wastewater. *ACS ES&T Water* **2023**, *3* (1), 118–128. DOI: 10.1021/acsestwater.2c00446.

(2) Chen, Y.-J.; Tang, J.-S.; Lin, A. Y.-C. Comprehensive nontargeted analysis of fluorosurfactant byproducts and reaction products in wastewater from semiconductor manufacturing. *Sustain. Environ. Res.* **2024**, *34*, 14. DOI: 10.1186/s42834-024-00221-1.

(3) Chen, Y. J.; Wang, R. D.; Shih, Y. L.; Chin, H. Y.; Lin, A. Y. Emerging perfluorobutane sulfonamido derivatives as a new trend of surfactants used in the semiconductor industry. *Environ. Sci. Technol.* **2024**, *58* (3), 1648–1658. DOI: 10.1021/acs.est.3c04435.

(4) Ober, C. K.; Käfer, F.; Deng, J. Y. Review of essential use of fluorochemicals in lithographic patterning and semiconductor processing. *J. Micro/Nanopatterning, Mater., Metrol.* **2022**, *21* (1), 010901. DOI: 10.1117/1.Jmm.21.1.010901.

(5) Osawa, Y.; Watanabe, S.; Takemura, K.; Nagura, S.; Tanaka, H.; Kawai, Y. New sulfonium salt and chemical amplification positive resist material. Japan Patent Office, JP3918881 B2, 2007.

(6) Kodama, K.; Sato, K.; Fujimori, T. Positive photosensitive composition. United States Patent, US6927009 B2, 2001.

(7) Ayothi, R.; Chang, S. W.; Felix, N.; Cao, H. B.; Deng, H.; Yueh, W.; Ober, C. K. New PFOS free photoresist systems for EUV lithography. *J. Photopolym. Sci. Technol.* **2006**, *19* (4), 515−520. DOI: 10.2494/photopolymer.19.515.

(8) Kawakami, A.; Utsumi, Y.; Matsuzawa, K.; Hirano, I.; Shimizu, H. Resist composition, resist pattern-forming method, compound, and acid generator. Japan Patent Office, JP5542402 B2, 2014.

(9) Padmanaban, M.; Dammel, R.; Lee, S.; Kim, W.-K.; Kudo, T.; McKenzie, D.; Rahman, D. Performance of imide and methide onium PAGs in 193-nm resist formulations. *Proc. SPIE, Advances in Resist Technology and Processing XX* **2003**, *5039*. DOI: 10.1117/12.487738.

(10) Fujiwara, T.; Ohashi, M.; Taniguchi, R. Sulfonium salt, resist composition, and patterning process. United States Patent, US10180626 B2, 2019.

(11) Namgung, R.; Park, H.; Kim, M.; Song, D.; Chon, M.; Kim, J. S.; Kim, H.-W.; Song, H.-J.; Choi, Y. J.; Hong, S.-K. Resist topcoat composition, and method of foraming patterns using the composition. United States Patent, US20230024422 A1, 2023.

(12) Iwashima, C.; Imai, G.; Okamura, H.; Tsunooka, M.; Shirai, M. Synthesis of i- and g-line sensitive photoacid generators and their application to photopolymer systems. *J. Photopolym. Sci. Technol.* **2003**, *16* (1), 91–96. DOI: 10.2494/photopolymer.16.91.

(13) Zhang, L.; Feng, B.; Pang, S.; Xin, H.; Li, K.; Jin, Y. Synthesis and performance study of nonionic photoacid generators based on Norbornene-imide. *J. Mol. Struct.* **2024**, *1304*, 137653. DOI: 10.1016/j.molstruc.2024.137653.

(14) Lee, Y. B.; Cho, H. Y.; Zhang, Y.; Kunz, M. A combination of NIT derivatives with sensitizers. European Patent Application, EP3182203 A1, 2017.

(15) Zhang, Y.; Greene, D.; Sharma, R. B. Sulfonic acid derivative compounds as photoacid generators in resist applications. United States Patent, US9709886 B2, 2017.

(16) Sanders, D. P. Advances in patterning materials for 193 nm immersion lithography. *Chem. Rev.* **2010**, *110* (1), 321–360. DOI: 10.1021/cr900244n.

(17) Neuwald, I. J.; Zahn, D.; Knepper, T. P. Are (fluorinated) ionic liquids relevant environmental contaminants? High-resolution mass spectrometric screening for per- and polyfluoroalkyl substances in environmental water samples led to the detection of a fluorinated ionic liquid. *Anal. Bioanal. Chem.* **2020**, *412* (20), 4881–4892. DOI: 10.1007/s00216-020-02606-8.

(18) Glüge, J.; Scheringer, M.; Cousins, I. T.; DeWitt, J. C.; Goldenman, G.; Herzke, D.; Lohmann, R.; Ng, C. A.; Trier, X.; Wang, Z. Y. An overview of the uses of per- and polyfluoroalkyl substances (PFAS). *Environ. Sci. Process. Impacts* **2020**, *22* (12), 2345–2373. DOI: 10.1039/d0em00291g.

(19) Guelfo, J. L.; Ferguson, P. L.; Beck, J.; Chernick, M.; Doria-Manzur, A.; Faught, P. W.; Flug, T.; Gray, E. P.; Jayasundara, N.; Knappe, D. R. U.; et al. Lithium-ion battery components are at the nexus of sustainable energy and environmental release of per- and polyfluoroalkyl substances. *Nat. Commun.* **2024**, *15* (1), 5548. DOI: 10.1038/s41467-024-49753-5.

(20) Scott, B. F.; De Silva, A. O.; Spencer, C.; Lopez, E.; Backus, S. M.; Muir, D. C. G. Perfluoroalkyl acids in Lake Superior water: Trends and sources. *J. Great Lakes Res.* **2010**, *36* (2), 277−284. DOI: 10.1016/j.jglr.2010.03.003.

(21) Scott, B. F.; Mactavish, D.; Spencer, C.; Strachan, W. M. J.; Muir, D. C. G. Haloacetic acids in Canadian lake waters and precipitation. *Environ. Sci. Technol.* **2000**, *34* (20), 4266−4272. DOI: 10.1021/es9908523.

(22) Scott, B. F.; Spencer, C.; Mabury, S. A.; Muir, D. C. G. Poly and perfluorinated carboxylates in north American precipitation. *Environ. Sci. Technol.* **2006**, *40* (23), 7167−7174. DOI: 10.1021/es061403n.

(23) Scott, B. F.; Spencer, C.; Martin, J. W.; Barra, R.; Bootsma, H. A.; Jones, K. C.; Johnston, A. E.; Muir, D. C. G. Comparison of haloacetic acids in the environment of the northern and southern hemispheres. *Environ. Sci. Technol.* **2005**, *39* (22), 8664−8670. DOI: 10.1021/es050118l.

(24) Scott, B. F.; Spencer, C.; Marvin, C. H.; MacTavish, D. C.; Muir, D. C. G. Distribution of haloacetic acids in the water columns of the Laurentian Great Lakes and Lake Malawi. *Environ. Sci. Technol.* **2002**, *36* (9), 1893−1898. DOI: 10.1021/es011156h.

(25) Neuwald, I. J.; Hübner, D.; Wiegand, H. L.; Valkov, V.; Borchers, U.; Nödler, K.; Scheurer, M.; Hale, S. E.; Arp, H. P. H.; Zahn, D. Ultra-short-chain PFASs in the sources of German drinking water: Prevalent, overlooked, difficult to remove, and unregulated. *Environ. Sci. Technol.* **2022**, *56* (10), 6380–6390. DOI: 10.1021/acs.est.1c07949.

(26) Janda, J.; Nödler, K.; Brauch, H. J.; Zwiener, C.; Lange, F. T. Robust trace analysis of polar (C_2-C_8) perfluorinated carboxylic acids by liquid chromatography-tandem mass spectrometry: method development and application to surface water, groundwater and drinking water. *Environ. Sci. Pollut. Res.* **2019**, *26* (8), 7326–7336. DOI: 10.1007/s11356-018-1731-x.

(27) Taniyasu, S.; Kannan, K.; Yeung, L. W. Y.; Kwok, K. Y.; Lam, P. K. S.; Yamashita, N. Analysis of trifluoroacetic acid and other short-chain perfluorinated acids (C_2-C_4) in precipitation by liquid chromatography-tandem mass spectrometry: Comparison to patterns of long-chain perfluorinated acids (C_5-C_{18}). *Anal. Chim. Acta* **2008**, *619* (2), 221–230. DOI: 10.1016/j.aca.2008.04.064.

(28) Yeung, L. W. Y.; Stadey, C.; Mabury, S. A. Simultaneous analysis of perfluoroalkyl and polyfluoroalkyl substances including ultrashort-chain C2 and C3 compounds in rain and river water samples by ultra performance convergence chromatography. *J. Chromatogr. A* **2017**, *1522*, 78–85. DOI: 10.1016/j.chroma.2017.09.049.

(29) Jiao, E. M.; Larsson, P.; Wang, Q.; Zhu, Z. L.; Yin, D. Q.; Kärrman, A.; Van Hees, P.; Karlsson, P.; Qiu, Y. L.; Yeung, L. W. Y. Further insight into extractable (organo)fluorine mass balance analysis of tap water from Shanghai, China. *Environ. Sci. Technol.* **2023**, *57* (38), 14330–14339. DOI: 10.1021/acs.est.3c02718.

(30) Ghorbani Gorji, S.; Mackie, R.; Prasad, P.; Knight, E. R.; Qu, X.; Vardy, S.; Bowles, K.; Higgins, C. P.; Thomas, K. V.; Kaserzon, S. L. Occurrence of ultrashort-chain PFASs in Australian environmental water samples. *Environ. Sci. Technol. Lett.* **2024**, (12), 1362–1369. DOI: 10.1021/acs.estlett.4c00750.

(31) Köke, N.; Zahn, D.; Knepper, T. P.; Frömel, T. Multi-layer solid-phase extraction and evaporation-enrichment methods for polar organic chemicals from aqueous matrices. *Anal. Bioanal. Chem.* **2018**, *410* (9), 2403−2411. DOI: 10.1007/s00216-018-0921-1.

(32) Zahn, D.; Frömel, T.; Knepper, T. P. Halogenated methanesulfonic acids: A new class of organic micropollutants in the water cycle. *Water Res.* **2016**, *101*, 292−299. DOI: 10.1016/j.watres.2016.05.082.

(33) Montes, R.; Rodil, R.; Placer, L.; Wilms, J. M.; Cela, R.; Quintana, J. B. Applicability of mixed-mode chromatography for the simultaneous analysis of C_1–C_{18} perfluoroalkylated substances. *Anal. Bioanal. Chem.* **2020**, *412* (20), 4849–4856. DOI: 10.1007/s00216-020-02434-w.

(34) Björnsdotter, M. K.; Yeung, L. W. Y.; Kärrman, A.; Jogsten, I. E. Ultra-short-chain perfluoroalkyl acids including trifluoromethane sulfonic acid in water connected to known and suspected point sources in Sweden. *Environ. Sci. Technol.* **2019**, *53* (19), 11093–11101. DOI: 10.1021/acs.est.9b02211.

(35) Mok, S.; Lee, S.; Choi, Y.; Jeon, J.; Kim, Y. H.; Moon, H. B. Target and non-target analyses of neutral per- and polyfluoroalkyl substances from fluorochemical industries using GC-MS/MS and GC-

TOF: Insights on their environmental fate. *Environ. Int.* **2023**, *182*, 108311. DOI: 10.1016/j.envint.2023.108311.

(36) Ayala-Cabrera, J. F.; Santos, F. J.; Moyano, E. Negative-ion atmospheric pressure ionisation of semi-volatile fluorinated compounds for ultra-high-performance liquid chromatography tandem mass spectrometry analysis. *Anal. Bioanal. Chem.* **2018**, *410* (20), 4913−4924. DOI: 10.1007/s00216-018-1138-z.

(37) Taniyasu, S.; Yeung, L.W.Y.; Lin, H.; Yamazaki, E.; Eun, H.; Lam, P.K.S.; Yamashita, N. Quality assurance and quality control of solid phase extraction for PFAS in water and novel analytical techniques for PFAS analysis. *Chemosphere* **2022**, *288*, 132440. DOI: 10.1016/j.chemosphere.2021.132440.

(38) Habib, A.; Noriega Landa, E.; Holbrook, K.; Walker, W.; Lee, W.-Y. Rapid, efficient, and green analytical technique for determination of fluorotelomer alcohol in water by stir bar sorptive extraction. *Chemosphere* **2023**, *338*, 139439. DOI: 10.1016/j.chemosphere.2023.139439

(39) Wang, N.; Szostek, B.; Buck, R. C.; Folsom, P. W.; Sulecki, L. M.; Capka, V.; Berti, W. R.; Gannon, J. T. Fluorotelomer alcohol biodegradation - Direct evidence that perfluorinated carbon chains breakdown. *Environ. Sci. Technol.* **2005**, *39* (19), 7516−7528. DOI: 10.1021/es0506760.

(40) Dimzon, I. K.; Westerveld, J.; Gremmel, C.; Frömel, T.; Knepper, T. P.; de Voogt, P. Sampling and simultaneous determination of volatile per- and polyfluoroalkyl substances in wastewater treatment plant air and water. *Anal. Bioanal. Chem.* **2017**, *409* (5), 1395−1404. DOI: 10.1007/s00216-016-0072-1.

(41) Bach, C.; Boiteux, V.; Hemard, J.; Colin, A.; Rosin, C.; Munoz, J. F.; Dauchy, X. Simultaneous determination of perfluoroalkyl iodides, perfluoroalkane sulfonamides, fluorotelomer alcohols, fluorotelomer iodides and fluorotelomer acrylates and methacrylates in water and sediments using solid-phase microextraction-gas chromatography/mass spectrometry. *J. Chromatogr. A* **2016**, *1448*, 98−106. DOI: 10.1016/j.chroma.2016.04.025.

(42) Dinglasan, M. J. A.; Ye, Y.; Edwards, E. A.; Mabury, S. A. Fluorotelomer alcohol biodegradation yields poly- and perfluorinated acids. *Environ. Sci. Technol.* **2004**, *38* (10), 2857−2864. DOI: 10.1021/es0350177.

(43) Koc, M.; Donten, M. A.; Musijowski, J.; Guo, X.; Fauland, A.; Lankmayr, E.; Trojanowicz, M. Application of gas chromatography to determination of Total Organic Fluorine after defluorination of perfluorooctanoic acid as a model compound. *Croat. Chem. Acta* **2011**, *84* (3), 399−406. DOI: 10.5562/cca1798.

(44) Dufkova, V.; Cabala, R.; Maradova, D.; Sticha, M. A fast derivatization procedure for gas chromatographic analysis of perfluorinated organic acids. *J. Chromatogr. A* **2009**, *1216* (49), 8659−8664. DOI: 10.1016/j.chroma.2009.10.042.

(45) Ayala-Cabrera, J. F.; Moyano, E.; Santos, F. J. Gas chromatography and liquid chromatography coupled to mass spectrometry for the determination of fluorotelomer olefins, fluorotelomer alcohols,

perfluoroalkyl sulfonamides and sulfonamido-ethanols in water. *J. Chromatogr. A* **2020**, *1609*, 460463. DOI: 10.1016/j.chroma.2019.460463.

(46) Rewerts, J.; Morre, J.; Massey Simonich, S.; Field, J. In-vial extraction large volume gas chromatography mass spectrometry for analysis of volatile PFASs on papers and textiles. *Environ. Sci. Technol.* **2018**, *52* (18), 10609–10616. DOI: 10.1021.acs.est.8b04304.

(47) Martin, J. W.; Muir, D. C.; Moody, C. A.; Ellis, D. A.; Kwan, W. C.; Solomon, K. R.; Mabury, S. A. Collection of airborne fluorinated organics and analysis by gas chromatography/chemical ionization mass spectrometry. *Anal. Chem.* **2002**, *74* (3), 584−590. DOI: 10.1021/ac015630d.

(48) Ayala-Cabrera, J. F.; Contreras-Llin, A.; Moyano, E.; Santos, F. J. A novel methodology for the determination of neutral perfluoroalkyl and polyfluoroalkyl substances in water by gas chromatography-atmospheric pressure photoionisation-high resolution mass spectrometry. *Anal. Chim. Acta* **2020**, *1100*, 97−106. DOI: 10.1016/j.aca.2019.12.004.

(49) Ruan, T.; Lin, Y.; Wang, T.; Jiang, G.; Wang, N. Methodology for studying biotransformation of polyfluoroalkyl precursors in the environment. *TrAC, Trends Anal. Chem.* **2015**, *67*, 167−178. DOI: 10.1016/j.trac.2014.11.017.

(50) Berger, U.; Langlois, I.; Oehme, M.; Kallenborn, R. Comparison of three types of mass spectrometers for HPLC/MS analysis of perfluoroalkylated substances and fluorotelomer alcohols. *Eur. J. Mass Spectrom.* **2004**, *10* (5), 579−588. DOI: 10.1255/ejms.679.

(51) Chu, S.; Letcher, R. J. Analysis of fluorotelomer alcohols and perfluorinated sulfonamides in biotic samples by liquid chromatography-atmospheric pressure photoionization mass spectrometry. *J. Chromatogr. A* **2008**, *1215* (1−2), 92−99. DOI: 10.1016/j.chroma.2008.10.103.

(52) Jacob, P.; Barzen-Hanson, K. A.; Helbling, D. E. Target and nontarget analysis of per- and polyfluoralkyl substances in wastewater from electronics fabrication facilities. *Environ. Sci. Technol.* **2021**, *55* (4), 2346–2356. DOI: 10.1021/acs.est.0c06690.

(53) Nason, S. L.; Koelmel, J.; Zuverza-Mena, N.; Stanley, C.; Tamez, C.; Bowden, J. A.; Godri Pollitt, K. J. Software comparison for nontargeted analysis of PFAS in AFFF-contaminated soil. *J. Am. Soc. Mass Spectrom.* **2021**, *32* (4), 840–846. DOI: 10.1021/jasms.0c00261.

(54) Zweigle, J.; Bugsel, B.; Fabregat-Palau, J.; Zwiener, C. PFΔScreen: An open-source tool for automated PFAS feature prioritization in non-target HRMS data. *Anal. Bioanal. Chem.* **2023**, *416*, 349–362. DOI: 10.1007/s00216-023-05070-2.

(55) Zweigle, J.; Bugsel, B.; Zwiener, C. FindPFΔS: Non-target screening for PFAS horizontal line Comprehensive data mining for MS^2 fragment mass differences. *Anal. Chem.* **2022**, *94* (30), 10788–10796. DOI: 10.1021/acs.analchem.2c01521.

(56) Helmus, R.; van de Velde, B.; Brunner, A. M.; ter Laak, T. L.; van Wezel, A. P.; Schymanski, E. L. patRoon 2.0: Improved non-target analysis workflows including automated transformation product screening. *J. Open Source Softw.* **2022**, *7* (71), 4029. DOI: 10.21105/joss.04029.

(57) Helmus, R.; Ter Laak, T. L.; van Wezel, A. P.; de Voogt, P.; Schymanski, E. L. patRoon: Open source software platform for environmental mass spectrometry based non-target screening. *J. Cheminformatics* **2021**, *13* (1), 1. DOI: 10.1186/s13321-020-00477-w.

(58) Houtz, E. F.; Sedlak, D. L. Oxidative conversion as a means of detecting precursors to perfluoroalkyl acids in urban runoff. *Environ. Sci. Technol.* **2012**, *46* (17), 9342–9349. DOI: 10.1021/es302274g.

(59) Houtz, E. F.; Higgins, C. P.; Field, J. A.; Sedlak, D. L. Persistence of perfluoroalkyl acid precursors in AFFF-impacted groundwater and soil. *Environ. Sci. Technol.* **2013**, *47* (15), 8187–8195. DOI: 10.1021/es4018877.

(60) Zhang, C.; Hopkins, Z. R.; McCord, J.; J., S. M.; Knappe, D. R. U. Fate of per- and polyfluoroalkyl ether acids in the total oxidizable precursor assay and implications for the analysis of impacted water. *Environ. Sci. Technol. Lett.* **2019**, *6* (11), 662–668. DOI: 10.1021/acs.estlett.9b00525.

(61) Ruyle, B. J.; Thackray, C. P.; McCord, J. P.; Strynar, M. J.; Mauge-Lewis, K. A.; Fenton, S. E.; Sunderland, E. M. Reconstructing the composition of per- and polyfluoroalkyl substances in contemporary aqueous film-forming foams. *Environ. Sci. Technol. Lett.* **2021**, *8* (1), 59–65. DOI: 10.1021/acs.estlett.0c00798.

(62) Baqar, M.; Zhaeo, M.; Saleem, R.; Cheng, Z.; Fang, B.; Dong, X. Y.; Chen, H.; Yao, Y. M.; Sun, H. W. Identification of emerging per- and polyfluoroalkyl subustances (PFAS) in E-waste recycling practices and new precursors for trifluoroacetic acid. *Environ. Sci. Technol.* **2024**, *58* (36), 16153–16163. DOI: 10.1021/acs.est.4c05646.

(63) Tsou, K.; Antell, E.; Duan, Y. H.; Olivares, C. I.; Yi, S.; Alvarez-Cohen, L.; Sedlak, D. L. Improved total oxidizable precursor assay for quantifying polyfluorinated compounds amenable to oxidative conversion to perfluoroalkyl carboxylic acids. *ACS ES&T Water* **2023**, *3* (9), 2996–3003. DOI: 10.1021/acsestwater.3c00224.

(64) Janda, J.; Nödler, K.; Scheurer, M.; Happel, O.; Nurenberg, G.; Zwiener, C.; Lange, F. T. Closing the gap – inclusion of ultrashort-chain perfluoroalkyl carboxylic acids in the total oxidizable precursor (TOP) assay protocol. *Environ. Sci. Process. Impacts* **2019**, *21* (11), 1926–1935. DOI: 10.1039/C9EM00169G.

(65) Martin, D.; Munoz, G.; Mejia-Avendaño, S.; Duy, S. V.; Yao, Y.; Volchek, K.; Brown, C. E.; Liu, J. X.; Sauvé, S. Zwitterionic, cationic, and anionic perfluoroalkyl and polyfluoroalkyl substances integrated into total oxidizable precursor assay of contaminated groundwater. *Talanta* **2019**, *195*, 533−542. DOI: 10.1016/j.talanta.2018.11.093.

(66) Houtz, E. F.; Sutton, R.; Park, J. S.; Sedlak, M. Poly- and perfluoroalkyl substances in wastewater: Significance of unknown precursors, manufacturing shifts, and likely AFFF impacts. *Water Res.* **2016**, *95*, 142–149. DOI: 10.1016/j.watres.2016.02.055.

(67) Forster, A. L. B.; Zhang, Y.; Westerman, D. C.; Richardson, S. D. Improved total organic fluorine methods for more comprehensive measurement of PFAS in industrial wastewater, river water, and air. *Water Res.* **2023**, *235*, 119859. DOI: 10.1016/j.watres.2023.119859.

(68) Nxumalo, T.; Akhdhar, A.; Mueller, V.; Simon, F.; von der Au, M.; Cossmer, A.; Pfeifer, J.; Krupp, E. M.; Meermann, B.; Kindness, A.; et al. EOF and target PFAS analysis in surface waters affected by sewage treatment effluents in Berlin, Germany. *Anal. Bioanal. Chem.* **2023**, *415*, 1195–1204. DOI: 10.1007/s00216-022-04500-x.

(69) Miaz, L. T.; Plassmann, M. M.; Gyllenhammar, I.; Bignert, A.; Sandblom, O.; Lignell, S.; Glynn, A.; Benskin, J. P. Temporal trends of suspect- and target-per/polyfluoroalkyl substances (PFAS), extractable organic fluorine (EOF) and total fluorine (TF) in pooled serum from first-time mothers in Uppsala, Sweden, 1996–2017. *Environ. Sci. Process. Impacts* **2020**, *22* (4), 1071−1083. DOI: 10.1039/C9EM00502A.

(70) Miyake, Y.; Yamashita, N.; Rostkowski, P.; So, M. K.; Taniyasu, S.; Lam, P. K. S.; Kannan, K. Determination of trace levels of total fluorine in water using combustion ion chromatography for fluorine: A mass balance approach to determine individual perfluorinated chemicals in water. *J. Chromatogr. A* **2007**, *1143* (1–2), 98–104. DOI: 10.1016/j.chroma.2006.12.071.

(71) Gehrenkemper, L.; Simon, F.; Roesch, P.; Fischer, E.; von der Au, M.; Pfeifer, J.; Cossmer, A.; Wittwer, P.; Vogel, C.; Simon, F. G.; et al. Determination of organically bound fluorine sum parameters in river water samples-comparison of combustion ion chromatography (CIC) and high resolution-continuum source-graphite furnace molecular absorption spectrometry (HR-CS-GFMAS). *Anal. Bioanal. Chem.* **2021**, *413* (1), 103–115. DOI: 10.1007/s00216-020-03010-y.

(72) US EPA. *Method 1621: Determination of adsorbable organic fluorine (AOF) in aqueous matrices by combustion ion chromatography (CIC)*; EPA 821-D-22-002; U.S. Environmental Protection Agency, Washington, DC, US, 2022.

(73) Wagner, A.; Raue, B.; Brauch, H.-J.; Worch, E.; Lange, F. T. Determination of adsorbable organic fluorine from aqueous environmental samples by adsorption to polystyrene-divinylbenzene based activated carbon and combustion ion chromatography. *J. Chromatogr. A* **2013**, *1295*, 82–89. DOI: 10.1016/j.chroma.2013.04.051.

(74) Willach, S.; Brauch, H.-J.; Lange, F. T. Contribution of selected perfluoroalkyl and polyfluoroalkyl substances to the adsorbable organically bound fluorine in German rivers and in a highly contaminated groundwater. *Chemosphere* **2016**, *145*, 342–350. DOI: 10.1016/j.chemosphere.2015.11.113.

(75) Han, Y. L.; Pulikkal, V. F.; Sun, M. Comprehensive validation of the adsorbable organic fluorine analysis and performance comparison of current methods for total per- and polyfluoroalkyl substances in water samples. *ACS ES&T Water* **2021**, *1* (6), 1474–1482. DOI: 10.1021/acsestwater.1c00047.

(76) von Abercron, E.; Falk, S.; Stahl, T.; Georgii, S.; Hamscher, G.; Brunn, H.; Schmitz, F. Determination of adsorbable organically bound fluorine (AOF) and adsorbable organically bound halogens as sum parameters in aqueous environmental samples using combustion ion chromatography (CIC). *Sci. Total Environ.* **2019**, *673*, 384–391. DOI: 10.1016/j.scitotenv.2019.04.068.

(77) Jacob, P.; Helbling, D. E. Exploring the evolution of organofluorine-containing compounds during simulated photolithography experiments. *Environ. Sci. Technol.* **2023**, *57* (34), 12819–12828. DOI: 10.1021/acs.est.3c03410.

(78) Ritter, E. E.; Dickinson, M. E.; Harron, J. P.; Lunderberg, D. M.; DeYoung, P. A.; Robel, A. E.; Field, J. A.; Peaslee, G. F. PIGE as a screening tool for per- and polyfluorinated substances in papers and textiles. *Nuclear Instruments & Methods in Physics Research Section B: Beam Interactions with Materials and Atoms* **2017**, *407*, 47–54. DOI: 10.1016/j.nimb.2017.05.052.

(79) Tighe, M.; Jin, Y. K.; Whitehead, H. D.; Hayes, K.; Lieberman, M.; Pannu, M.; Plumlee, M. H.; Peaslee, G. F. Screening for per- and polyfluoroalkyl substances in water with particle induced gamma-ray emission spectroscopy. *ACS ES&T Water* **2021**, *1* (12), 2477–2484. DOI: 10.1021/acsestwater.1c00215.
